# Vitamin K Supplementation in Postmenopausal Women with Osteopenia (ECKO Trial): A Randomized Controlled Trial

**DOI:** 10.1371/journal.pmed.0050196

**Published:** 2008-10-14

**Authors:** Angela M Cheung, Lianne Tile, Yuna Lee, George Tomlinson, Gillian Hawker, Judy Scher, Hanxian Hu, Reinhold Vieth, Lilian Thompson, Sophie Jamal, Robert Josse

**Affiliations:** 1 Division of General Internal Medicine, Department of Medicine, University of Toronto, Toronto, Ontario, Canada; 2 Division of Endocrinology, Department of Medicine, University of Toronto, Toronto, Ontario, Canada; 3 Clinical Epidemiology Program, Department of Health Policy, Management and Evaluation, University of Toronto, Toronto, Ontario, Canada; 4 Osteoporosis and Women's Health Programs, University Health Network, Toronto, Ontario, Canada; 5 Division of Rheumatology, Department of Medicine, University of Toronto, Toronto, Ontario, Canada; 6 Department of Pathology and Laboratory Medicine, University of Toronto, Toronto, Ontario, Canada; 7 Department of Nutritional Sciences, University of Toronto, Toronto, Ontario, Canada; The University of York, United Kingdom

## Abstract

**Background:**

Vitamin K has been widely promoted as a supplement for decreasing bone loss in postmenopausal women, but the long-term benefits and potential harms are unknown. This study was conducted to determine whether daily high-dose vitamin K1 supplementation safely reduces bone loss, bone turnover, and fractures.

**Methods and Findings:**

This single-center study was designed as a 2-y randomized, placebo-controlled, double-blind trial, extended for earlier participants for up to an additional 2 y because of interest in long-term safety and fractures. A total of 440 postmenopausal women with osteopenia were randomized to either 5 mg of vitamin K1 or placebo daily. Primary outcomes were changes in BMD at the lumbar spine and total hip at 2 y. Secondary outcomes included changes in BMD at other sites and other time points, bone turnover markers, height, fractures, adverse effects, and health-related quality of life. This study has a power of 90% to detect 3% differences in BMD between the two groups. The women in this study were vitamin D replete, with a mean serum 25-hydroxyvitamin D level of 77 nmol/l at baseline. Over 2 y, BMD decreased by −1.28% and −1.22% (*p* = 0.84) (difference of −0.06%; 95% confidence interval [CI] −0.67% to 0.54%) at the lumbar spine and −0.69% and −0.88% (*p* = 0.51) (difference of 0.19%; 95% CI −0.37% to 0.75%) at the total hip in the vitamin K and placebo groups, respectively. There were no significant differences in changes in BMD at any site between the two groups over the 2- to 4-y period. Daily vitamin K1 supplementation increased serum vitamin K1 levels by 10-fold, and decreased the percentage of undercarboxylated osteocalcin and total osteocalcin levels (bone formation marker). However, C-telopeptide levels (bone resorption marker) were not significantly different between the two groups. Fewer women in the vitamin K group had clinical fractures (nine versus 20, *p* = 0.04) and fewer had cancers (three versus 12, *p* = 0.02). Vitamin K supplements were well-tolerated over the 4-y period. There were no significant differences in adverse effects or health-related quality of life between the two groups. The study was not powered to examine fractures or cancers, and their numbers were small.

**Conclusions:**

Daily 5 mg of vitamin K1 supplementation for 2 to 4 y does not protect against age-related decline in BMD, but may protect against fractures and cancers in postmenopausal women with osteopenia. More studies are needed to further examine the effect of vitamin K on fractures and cancers.

**Trial registration:** ClinicalTrials.gov (#NCT00150969) and Current Controlled Trials (#ISRCTN61708241)

## Abstract 2

Angela Cheung and colleagues investigate whether vitamin K1 can prevent bone loss among postmenopausal women with osteopenia.

## Editors' Summary

**Background.:**

Osteoporosis is a bone disease in which the bones gradually become less dense and more likely to break. In the US, 10 million people have osteoporosis and 18 million have osteopenia, a milder condition that precedes osteoporosis. In both conditions, insufficient new bone is made and/or too much old bone is absorbed. Although bone appears solid and unchanging, very little bone in the human body is more than 10 y old. Old bone is continually absorbed and new bone built using calcium, phosphorous, and proteins. Because the sex hormones control calcium and phosphorous deposition in the bones and thus bone strength, the leading cause of osteoporosis in women is reduced estrogen levels after menopause. In men, an age-related decline in testosterone levels can cause osteoporosis. Most people discover they have osteoporosis only when they break a bone, but the condition can be diagnosed and monitored using bone mineral density (BMD) scans. Treatments can slow down or reverse bone loss (antiresorptive therapies) and some (bone formation therapies) can even make bone and build bone tissue.

**Why Was This Study Done?:**

Although regular exercise and a healthy diet can help to keep bones strong, other ways of preventing osteoporosis are badly needed. Recently, the lay media has promoted vitamin K supplements as a way to reduce bone loss in postmenopausal women. Vitamin K (which is found mainly in leafy green vegetables) is required for a chemical modification of proteins called carboxylation. This modification is essential for the activity of three bone-building proteins. In addition, there is some evidence that low bone density and fractures are associated with a low vitamin K intake. However, little is known about the long-term benefits or harms of vitamin K supplements. In this study, the researchers investigate whether a high-dose daily vitamin K supplement can safely reduce bone loss, bone turnover, and fractures in postmenopausal women with osteopenia in a randomized controlled trial called the “Evaluation of the Clinical Use of Vitamin K Supplementation in Post-Menopausal Women With Osteopenia” (ECKO) trial.

**What Did the Researchers Do and Find?:**

In the study, 440 postmenopausal women with osteopenia were randomized to receive 5mg of vitamin K1 (the type of vitamin K in North American food; the recommended daily adult intake of vitamin K1 is about 0.1 mg) or an inactive tablet (placebo) daily for 2 y; 261 of the women continued their treatment for 2 y to gather information about the long-term effects of vitamin K1 supplementation. All the women had regular bone density scans of their lower back and hips and were examined for fractures and for changes in bone turnover. After 2 y and after 4 y, lower back and hip bone density measurements had decreased by similar amounts in both treatment groups. The women who took vitamin K1 had 10-fold higher amounts of vitamin K1 in their blood than the women who took placebo and lower amounts of a bone formation marker; the levels of a bone resorption marker were similar in both groups. Over the 4-y period, fewer women in the vitamin K group had fractures (nine versus 20 women in the placebo group), and fewer had cancer (three versus 12). Finally, vitamin K supplementation was well tolerated over the 4-y period and adverse health effects were similar in the two treatment groups.

**What Do These Findings Mean?:**

These findings indicate that a high daily dose of vitamin K1 provides no protection against the age-related decline in bone density in postmenopausal women with osteopenia, but that vitamin K1 supplementation may protect against fractures and cancers in these women. The apparent contradiction between the effects of vitamin K1 on bone density and on fractures could mean that vitamin K1 supplements strengthen bone by changing factors other than bone density, e.g., by changing its fine structure rather than making it denser. However, because so few study participants had fractures, the difference in the fracture rate between the two treatment groups might have occurred by chance. Larger studies are therefore needed to examine the effect of vitamin K1 on fractures (and on cancer) and, until these are done, high-dose vitamin K1 supplementation should not be recommended for the prevention of osteoporosis.

**Additional Information.:**

Please access these Web sites via the online version of this summary at http://dx.doi.org/10.1371/journal.pmed.0050196.

- The US National Institute of Arthritis and Musculoskeletal and Skin Diseases provides detailed information about osteoporosis (in English and Spanish) and links to other resources, including an interactive web tool called Check Up On Your Bones
- MedlinePlus provides links to additional information about osteoporosis (in English and Spanish)
- The MedlinePlus Encyclopedia has a page about vitamin K
- The UK Food Standards Agency provides information about vitamin K
- Full details about the ECKO trial are available on the ClinicalTrials.gov Web site
- The Canadian Task Force for Preventive Health Care provides recommendations on the prevention of osteoporosis and osteoporotic fractures in postmenopausal women
- Osteoporosis Canada provides information on current topics related to osteoporosis

## Introduction

Vitamin K is best known for its function in the blood coagulation pathway, but recent data suggest that the K vitamins play an important role in bone metabolism, perhaps at serum levels higher than those required for normal blood coagulation. Vitamin K is the essential cofactor for the carboxylation of glutamate to gamma-carboxyglutamic acid (Gla), which confers functionality to vitamin K–dependent Gla-containing proteins. Three Gla-containing bone proteins, all synthesized by osteoblasts, have been identified: osteocalcin, matrix Gla protein, and protein S. The K vitamin family has five groups: vitamins K1 (phylloquinone) and K2 (menaquinones) occur naturally in foods, whereas K3, K4, and K5 are synthetic.

Over the past 5 y, vitamin K supplements have been widely promoted by the lay media for increasing bone mass. These supplements have also been touted to prevent heart disease and cancer [1,2]. Vitamin K2 is an approved treatment for osteoporosis in Japan. Low-dose vitamin K1 is currently available in health food stores and over the Internet across the United States and Canada. Although earlier epidemiologic studies showed low vitamin K1 intake and low plasma vitamin K1 levels to be associated with low bone mineral density (BMD) [3,4] and increased osteoporotic fracture risk in postmenopausal women [5–8], more recent randomized trials using low-dose vitamin K1 supplements showed conflicting results on BMD [9–12]. None has been designed to examine the effect of vitamin K1 supplementation on fractures. Thus, the question of whether daily vitamin K1 supplementation reduces bone loss or decreases fracture risk remains controversial.

We conducted a 2- to 4-y randomized, placebo-controlled, double-blind study for the Evaluation of the Clinical use of vitamin K supplementation in postmenopausal women with Osteopenia (the ECKO trial). We chose 5 mg of vitamin K1 daily because previous data suggest that 1 mg daily is needed to maximally carboxylate osteocalcin [13], that maximal carboxylation of matrix Gla protein may require more than 1 mg daily, and that 10 mg daily for more than 3 mo was safe with no significant adverse effects [14]. At the time of planning for this study, we were also aware of two other low-dose vitamin K1 supplementation trials—one using 1 mg [10] and the other using 500 μg [9] of vitamin K1 daily. We decided to use a much higher dose so that the results from our study would complement those from the other two studies. Our primary objectives were to determine the effects of daily vitamin K1 supplementation on BMDs at the lumbar spine and total hip at 2 y. Our hypothesis was that vitamin K1 supplementation would reduce bone loss in postmenopausal women at those sites over a 2-y period. Our secondary objectives were to investigate the effects of vitamin K1 supplementation on BMDs at the femoral neck and the ultradistal radius and at other time points, bone turnover markers, change in height, fractures, and long-term adverse effects on health and health-related quality of life.

## Methods

### Design Overview

This study was originally designed as a 2-y single-center, randomized, double-blind, placebo-controlled trial of daily oral supplementation of vitamin K1 (5 mg) versus placebo (see Text S1 and S3). Because of considerable interest over the past few years in the long-term safety and efficacy of vitamin K on fractures, we extended our study for up to 4 y of supplementation in women who joined the study earlier (see Text S2 and S3). Specifically, we invited all participants who started the study prior to 15 March 2004 to join the extension study for a duration of 6–24 mo, so that their final study visit date was no later than 30 September 2006.

We obtained an investigational new drug number from Health Canada on 31 December 2001 for this study. Postmenopausal women were recruited through health fairs, advertisements, and posters from January 2002 to September 2004. All women gave written informed consent prior to any study procedures. They were examined annually at the University Health Network according to International Committee on Harmonization (ICH) good clinical practice guidelines. Outcome and safety data collection were completed by 30 October 2006, and 2 March 2007, respectively, and unblinding occurred on 8 March 2007. Compliance was assessed by counting leftover pills at follow-up visits by two study staff members who were working independently and blinded to treatment assignment. Participants taking 80% or more of the prescribed study pills were considered compliant. The protocol was approved by the University Health Network (UHN) and the University of Toronto research ethics boards.

### Study Participants

We chose postmenopausal women with osteopenia as our study population because they are at increased risk of developing osteoporosis and fractures. Women were eligible to participate in the study if they were postmenopausal (at least 1 y after last menses) and had their lowest *t*-score (lumbar spine [L1–L4], total hip, or femoral neck) between −1.0 and −2.0. They were excluded if they had osteoporosis; a fragility fracture after age 40; any bone medication use in the past 3 mo (such as bisphosphonates, selective estrogen receptor modulators, hormone replacement therapy, or calcitonin); any bisphosphonate use for more than 6 mo; known metabolic bone diseases such as primary hyperparathyroidism or Paget disease; decompensated diseases of the liver, kidney, pancreas, lung, or heart; history of active cancer in the past 5 y; or chronic oral steroid or warfarin use. They were also excluded if they were on high doses of vitamin A (>10,000 IU/d) or E (>400 IU/d), as these interfere with vitamin K metabolism [15].

### Interventions and Randomization

Both vitamin K1 and placebo pills were manufactured by Tishcon to look and taste the same. The UHN research pharmacy labeled and dispensed study pills according to a computer-generated random number table in blocks of ten. Participants, study coordinators, investigators, and outcome assessors were all blinded to the assignment. Participants were asked to take their study pill in the morning with food. They were also given calcium and vitamin D supplements that approximated a total intake (diet plus supplements) of 1,500 mg of calcium and 800 IU of vitamin D per day.

### Outcomes and Follow-up

Primary outcome measures were percentage change in BMD at the lumbar spine (L1–L4) and total hip from baseline to 2 y. Secondary outcome measures included BMD at the femoral neck and ultradistal radius, bone formation (osteocalcin, OC) and resorption (C-terminal telopeptide, CTX) markers, fractures, height, percentage of undercarboxylated osteocalcin (ucOC) and serum vitamin K levels (measures of vitamin K status), adverse effects, and health-related quality of life.

BMD at the lumbar spine, total hip, femoral neck, and ultradistal radius were measured yearly, and vertebral fracture assessment (VFA) was performed at baseline, 24, and 48 mo, or at final visit. Tests were performed according to standard protocol using one Hologic 4500A densitometer at the UHN Bone Density Laboratory. Both the technologist and the BMD densitometrist (YL) were certified by the International Society of Clinical Densitometry, not involved in the care of study participants, and blinded to treatment assignment. Women were discontinued from the study if their annual BMD T-score at the lumbar spine, femoral neck, or total hip fell below −2.5; they were not allowed to participate in the study extension if their BMDs dropped more than 10% over 2 y. The coefficients of variation (CVs) for BMD at the lumbar spine and total hip were 0.89% and 1.09%, respectively. Using the valid and reliable Genant semiquantitative method for assessing morphometric vertebral fractures [16], vertebrae were graded as normal, Grade 1 (20%–25% decrease in height), Grade 2 (25%–40%), or Grade 3 (>40%). We defined a new morphometric fracture as a deformity occurring in a normal vertebra or a worsening grade of deformity during the study period.

Reported clinical fractures were confirmed by radiographs or radiological reports, adjudicated by two investigators (GH and RJ) independently and classified into fragility (sustained with minimal trauma such as falling from standing height) or nonfragility fractures. A third investigator (SJ) resolved differences in cases of discordance. The a priori fracture outcomes included all clinical fractures (except fingers and toes) and all new morphometric vertebral fractures. We also measured height using a Harpenden stadiometer and health-related quality of life using the Medical Outcomes Study 36-item Short-Form General Health Survey (SF-36) [17,18].

We asked our participants to inform us of any new medical condition, hospitalization, or symptom they experienced during the study. In addition, at every study visit (baseline, 3, 6, 12, 18, 24, 30, 36, 42, and 48 mo), participants were asked about their health and any new medications or change in medications during the intervening months. All these symptoms, medications, and conditions were collected in a standardized fashion. Serious adverse events (SAEs) are defined as any hospitalizations, cancer, life-threatening events, or death. We used the standard definition according to the ICH Guidelines for the Conduct of Clinical Trials. Adverse events (AEs) are defined as any untoward effect experienced by the participant that are not considered an SAE. All the AEs and SAEs were coded by a physician blinded to study group assignment using ICD-9 (International Statistical Classification of Diseases and Related Health Problems, ninth revision) codes. We requested backup medical records including pathological reports and death certificates for all SAEs, and these were adjudicated by two blinded physician reviewers. All SAEs and AEs with cumulative incidence of greater than 1% in either the vitamin K or the placebo group were analyzed to see if there were differences between groups.

Serum was collected in the morning after an overnight fast at baseline, 3, 12, 24, 36, and 48 mo, stored in a −80 °C freezer, and analyzed in batches at the end of the study. Aliquots for ucOC (treated with hydroxyapatite) [19] and OC were run side-by-side using the N-Mid Osteocalcin electrochemiluminescent assay, and CTX was measured using the B-Crosslaps assay (Elecsys 2010 Immunochemistry Auto-analyzer, Roche Diagnostics, Germany). Intra-assay and inter-assay variability were 0%–2.5% and 1%–2.5% for OC and 0.1%–3.5% and 2.2%–3.1% for CTX, respectively. The percentage of ucOC was expressed as the ratio of ucOC to OC multiplied by 100. Serum vitamin K levels were measured in the Tufts University Vitamin K laboratory using reversed-phase high-performance liquid chromatography with postcolumn, solid-phase chemical reduction of phylloquinone to its hydroquinone, followed by fluorometric detection [20]. The CV of the vitamin K1 assay was 5.3%. The estimated limits of detection for vitamin K1, menaquinone 4, and menaquinone 7 were 0.08 pmol, 0.07 pmol, and 0.40 pmol per injection, respectively. Serum 25-hydroxyvitamin D levels were measured in the UHN clinical laboratory using the radioimmunoassay method (Diasorin, Stillwater, MN). The inter-assay CV was 9.5%–11.5% over the study period.

### Statistical Analyses

All analyses in this article were prespecified and finalized by 30 November 2006, prior to any analysis and prior to unblinding on 8 March 2007. For our 2-y BMD analyses, we included all 440 women based on intention to treat using last observation carried forward for missing data. Analyses of all other outcomes, including comparisons of BMD beyond 2 y, used only observed values. The main test statistic was the two-sample *t*-test. In sensitivity analyses, we used analysis of covariance to control for any dependence of change on baseline values. We also analyzed our data according to compliance and serum vitamin K and 25-hydroxyvitamin D levels by looking for a statistical interaction between groups and using linear regression models with the various 2-y outcomes.

We used the log-rank test and survival analysis techniques to examine the effect of vitamin K supplementation on fracture and cancer incidence. Time was defined as number of days from randomization to the first endpoint (if one occurred), death, or date of last contact. Kaplan-Meier plots were used to graphically explore event-free survival. Cox proportional hazards models were used to compare the effects of vitamin K and placebo. We also investigated whether mean serum vitamin K levels over the first 2 y had any relationship to fracture or cancer incidence.

Based on preliminary estimates of standard deviations in BMD at the lumbar spine and total hip in postmenopausal women with osteopenia, we determined that 200 participants were required in each group for the study to have a statistical power of 80% to detect clinically meaningful differences of 3% between treatment groups while allowing for a projected 10% withdrawal and 5% lost to follow-up rate per year. We over-recruited to 440, resulting in a power of 90%.

## Results

### Baseline Characteristics

**Figure 1:**
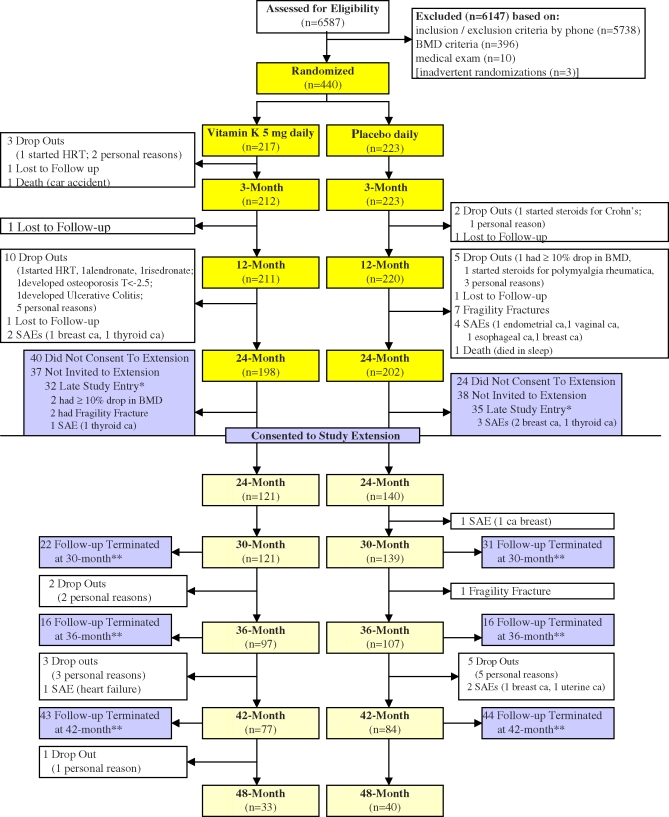
Flowchart of the ECKO Trial showing Participation of Women in the Study This flow diagram shows the number of study participants remaining in the study at each time point. *Women were not invited to participate in study extension if they were enrolled after 15 March 2004. **Participation was terminated for these women because the study was terminated. All final visits occurred between March and October 2006.

We screened 6,587 women and invited 848 to our center for a screening visit (Figure 1). Of those, 440 postmenopausal women with osteopenia who satisfied inclusion and exclusion criteria were randomly assigned to 5 mg of vitamin K1 daily (217 women) or placebo (223 women). Three others were inadvertently randomized (one woman with hyperthyroidism, one who was perimenopausal, and one with a small breast mass at screening that was subsequently diagnosed as breast cancer); these women were excluded from all analyses. Baseline characteristics of the two groups were similar (Table 1). Mean age of participants was 59 y (range 40 to 82), and 88% were European Canadian. Mean baseline serum 25-hydroxyvitamin D level was 77 nmol/l. At 2 y, 402 women (91.4%) had active follow-up, and 363 (83.2%) were compliant (82.5% in the vitamin K group and 83.9% in the placebo group). Of the women who joined the study prior to 15 March 2004, a total of 261 women (121 on vitamin K, 140 on placebo) consented to participate in the study extension. There was no difference in baseline characteristics between the two groups, and there was no difference in baseline characteristics between those who participated in the extension and those who did not. During the study extension, 87.5% (84.9% in the vitamin K group and 89.9% in the placebo group) and 88.6% (82.4% in the vitamin K group and 93.3% in the placebo group) were compliant at 3 and 4 y, respectively.

**Table 1:**
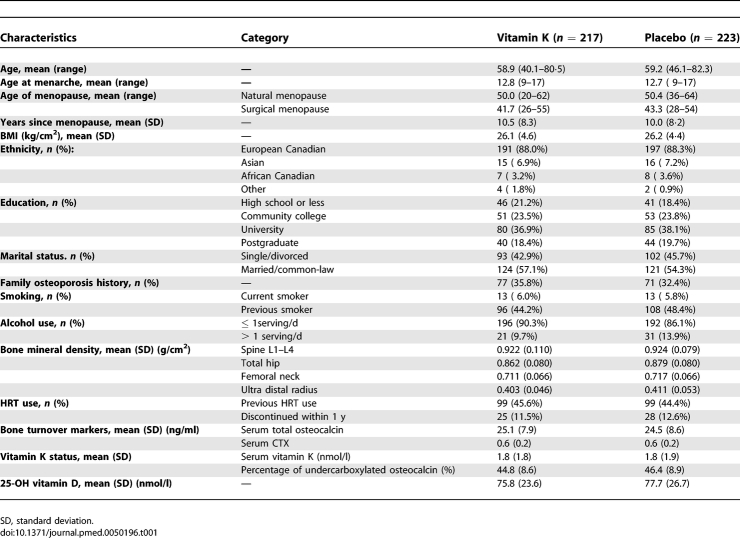
Baseline Characteristics of Women Participating in the ECKO Trial

### Bone Mineral Densities, Turnover Markers, and Fractures

**Figure 2:**
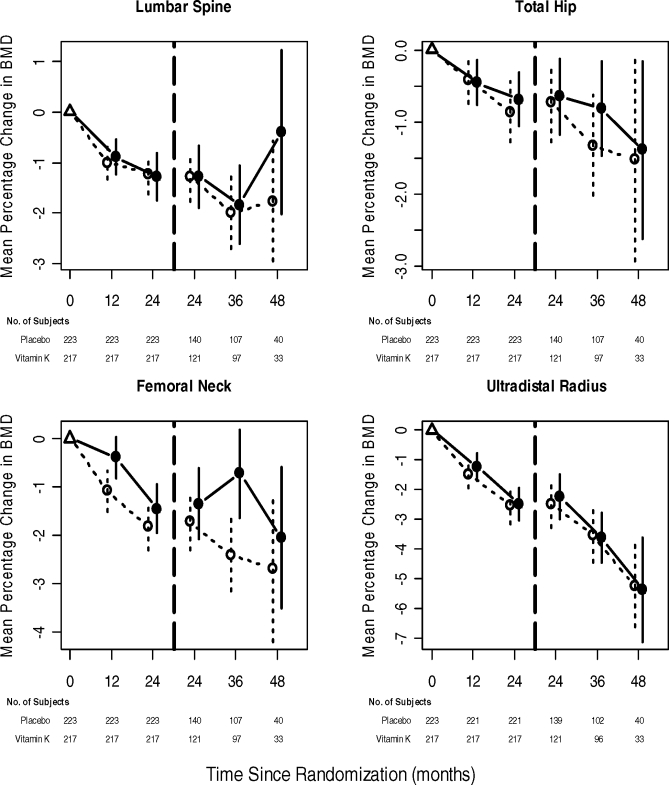
Changes in Bone Mineral Density over Time These graphs show mean percentage change from baseline (Δ) in bone mineral densities at the lumbar spine (L1–L4), total hip, femoral neck, and ultradistal radius sites in the vitamin K (•) and placebo (○) groups over time, with their respective 95% confidence intervals. From baseline to 2 y (left part of graphs), all 440 women were included in the analyses based on intention-to-treat principle. We used last observation carried forward for any missing data in later visits. The analyses of the 2- to 4-y outcomes in the study extension (right part of graphs) were based on observed outcomes only.

In the vitamin K and placebo groups, respectively, BMD decreased by −1.28% and −1.22% (*p* = 0.84) (difference of −0.06%; 95% confidence interval [CI], −0.67% to 0.54%) at the lumbar spine and −0.69% and −0.88% (*p* = 0.51) (difference of 0.19%; 95% CI −0.37% to 0.75%) at the total hip over 2 y. With the exception of a 1.7% difference in BMD at the femoral neck between the two groups at 36 mo, the changes in BMDs at the lumbar spine, total hip, femoral neck, and ultradistal radius were similar over the 2- to 4-y treatment period (Figure 2). Results did not change when we controlled for baseline BMD values or compliance.

**Figure 3:**
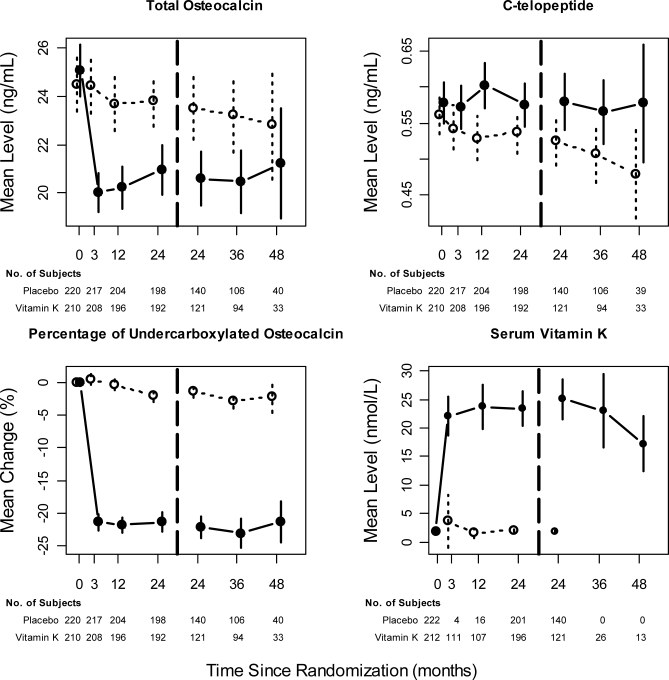
Changes in Biochemical Markers of Bone Turnover and Vitamin K Status Over Time These graphs showed mean total OC levels (bone formation marker), mean CTX (bone resorption marker), mean change in percentage of ucOC, and mean serum vitamin K levels in the vitamin K (•) and placebo (○) groups over time, with their respective 95% confidence intervals. All analyses were performed using observed outcomes. Because of limited study budget, we analyzed baseline and 2-y serum vitamin K levels in almost all the participants, but only a random sample at the other time points for those in the vitamin K group and none in the placebo group beyond 2 y.

At 2 y, serum total OC levels decreased (21 ng/ml versus 24 ng/ml, *p* < 0.0001) in women supplemented with vitamin K compared to placebo, but serum CTX levels were similar between groups (0.58 ng/ml versus 0.54 ng/ml, *p* = 0.07) (Figure 3). Although the difference in mean CTX levels between the two groups was statistically significant at 12 mo, the differences at other time points were not. In addition, most of these differences were not of clinically meaningful magnitudes.

**Figure 4:**
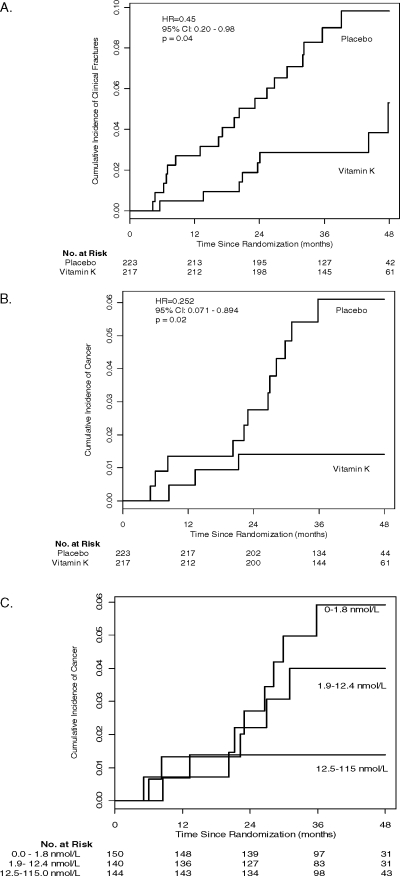
Cumulative Incidence of Clinical Fractures and Cancer The cumulative incidence of clinical fractures (A) and cancer (B) are shown for the vitamin K and placebo groups (C). The relationship of the cumulative incidence of cancer according to tertiles of 2-y serum vitamin K levels (shown in brackets and expressed in nmol/l). When Cox regression analysis was performed using the 2-y serum vitamin K levels as a continuous log-transformed variable, the relationship between vitamin K levels and the cumulative incidence of cancer was statistically significant at *p* < 0.05.

During the study and follow-up period, 32 clinical fractures were sustained by 29 women: nine women in the vitamin K group (three radius, one vertebra, one rib, three knees [tibial plateau, patella, subchondral], two feet [metatarsal and cuboid], and one ankle [tip of fibula]) and 20 women in the placebo group (nine radius, two humerus, one clavicle, one hip, one tibia, four foot, two ankles [bimalleolar, fibula], and one hand) (hazard ratio [HR] = 0.41, 95% CI 0.15 to 1.18, *p* = 0.08 at 2 y and HR = 0.45, 95% CI 0.20 to 0.98, *p* = 0.04 at 4 y) (Figure 4A). Of these 32 clinical fractures, 17 were considered fragility fractures (six fractures sustained by four women on vitamin K and 11 fractures sustained by ten women on placebo, *p* = 0.11). A total of 47 women had new morphometric vertebral fractures on VFA (most were Grade 1 fractures): 25 in the vitamin K group and 22 in the placebo group (*p* = 0.56). There was no difference in mean changes in height between groups.

### Relationship between Serum Vitamin K, Vitamin D levels, and BMD and Fractures

Daily 5 mg of vitamin K1 supplementation increased serum vitamin K1 levels (22.6 nmol/l versus 2.0 nmol/l, *p* < 0.0001 at 2 y) (Figure 3D), but did not increase circulating vitamin K2 (menaquinones 4 and 7) levels. Vitamin K1 supplementation decreased ucOC and percentage of ucOC (−52.8% versus −3.5%, *p* < 0.0001, and −21.4% versus −2.0%, *p* < 0.0001 at 2 y, respectively) (Figure 3C). Serum vitamin K1 and serum 25-hydroxyvitamin D levels were not related to percentage change in BMD, CTX, or fractures. There was also no relationship between these outcomes and the interaction of serum vitamin K1 and 25-hydroxyvitamin D levels.

### Adverse Effects and Health-Related Quality of Life

One or more adverse events were experienced by 384 women (87.3%) during the first 2 y of the study, but there were no significant differences between groups. Nausea and vomiting were reported in 11 women (5.1%) on vitamin K and ten women (4.5%) on placebo (*p* = 0.77). From 2 to 4 y, 69.6% (188/ 270) had one or more adverse event: 72.2% (91/126) in the vitamin K group and 67.4% (97/144) on placebo (*p* = 0.46).

SAEs occurred in 9.1% (40/440) of participants: 6.9% (15/217) in the vitamin K group and 11.2% (25/223) in the placebo group. These included hospitalizations for pneumonia, heart failure, gastrointestinal bleeding, elective and nonelective surgeries, cancer, and death. Cancer incidence was lower in the vitamin K group than in the placebo group (three versus 12, *p* = 0.02; HR = 0.25, 95% CI 0.07 to 0.89) (Figure 4B). Higher mean serum vitamin K levels over the duration of the study correlated with lower cancer incidence (*p* < 0.05) (Figure 4C). Because half of the cases were breast cancers (one in the vitamin K group and six in the placebo group), we calculated the Gail breast cancer risk score [21] from baseline data and found that there was no difference between the vitamin K and the placebo groups (1.70% versus 1.71% risk of having invasive breast cancer in the next 5 y). There were five deaths during the study and follow-up period: one woman on vitamin K (passenger in a car accident) and four women on placebo (three who died of cancers, and one who died in her sleep from arrhythmogenic right ventricular cardiomyopathy). There was no difference in health-related quality of life between groups.

## Discussion

Our study showed that 5 mg of vitamin K1 supplementation daily for 2–4 y did not protect against age-related decline in BMD at the lumbar spine, total hip, femoral neck, or ultradistal radius in postmenopausal women with osteopenia who were vitamin D replete. We also showed that vitamin K1 supplementation did not decrease bone resorption, but did protect against clinical fractures and cancers. Daily supplementation with high-dose vitamin K1 significantly increased serum vitamin K levels and decreased percentage of undercarboxylated osteocalcin. It was very well tolerated with no significant adverse effects.

Our BMD data are consistent with data from four recently published (or presented) randomized trials using much lower doses of vitamin K1 supplements [9–12], although the interpretation of the data differed somewhat among research groups. Booth and colleagues randomized 452 healthy men and women between 60 and 80 y of age to receive a multivitamin that contained either 500 μg/d of vitamin K1 or no vitamin K1 plus a daily calcium (600 mg) and vitamin D (400 IU) supplement [9]. After 3 y, they did not find any differences in BMD at the femoral neck, lumbar spine, or total body between the two groups. The Binkley group studied 381 postmenopausal women and found that neither vitamin K1 (1 mg/d) nor vitamin K2 (45 mg/d) supplementation for 2 y affected BMD at the lumbar spine or total hip [10].

In contrast, two earlier trials observed differences in the femoral neck and ultradistal radius sites. Braam and colleagues randomized 181 postmenopausal women between 50 and 60 y of age to a daily supplement containing placebo, minerals (calcium, magnesium, and zinc), and vitamin D (320 IU/d), or minerals and vitamins D and K1 (1 mg/d) [9]. After 3 y, the group receiving vitamin K had less bone loss at the femoral neck (BMD difference of 1.7%) but not at the lumbar spine. In the Bolton-Smith study [10], 244 postmenopausal women were randomized to receive placebo, vitamin K1 (200ug/d), vitamin D3 (400 IU) plus calcium (1,000 mg/d), or vitamins K1 and D plus calcium. After 2 y, there was no significant difference in BMD at any site between groups; however, the group taking the combined vitamins K, D, and calcium showed a 1.6% increase in BMD at the ultradistal radius site compared to baseline. Based on these data, the authors of these two trials concluded that vitamin K1 supplementation is helpful to prevent bone loss in postmenopausal women, especially when combined with vitamin D and calcium. We feel that the small differences observed in these two studies are likely due to measurement variability as well as statistical issues, and are of little clinical significance.

Our study showed that daily 5 mg of vitamin K1 supplementation increased serum vitamin K level by approximately 10-fold, but decreased serum ucOC, percentage of ucOC and total OC (a bone formation marker) levels. The decrease in ucOC and percentage of ucOC reflect improved vitamin K status. We do not fully understand the mechanism behind decreased serum total OC; however, we have two hypotheses. As more OC becomes carboxylated and functional with improved vitamin K status, less is needed, and thus less is synthesized and released into the circulation. The other potential explanation for this observation is that more functional OC will be bound to bone rather than circulating in the blood stream, thus lowering the serum total OC level. This decrease in total OC, as well as the decrease in percentage of ucOC, has been previously described by other groups [22,23].

Similar to data from recently published studies [9,10,22], vitamin K1 supplementation in our study did not result in a decrease in bone resorption. Our mean serum CTX levels were similar between the two groups at all time points except at 12 mo. The difference observed at 12 mo is likely by chance, as the differences at most other time points are not of clinically significant magnitudes.

Despite a lack of effect on BMD and bone resorption, we observed a lower incidence of clinical fractures among women in the vitamin K1 group, suggesting that the effect of vitamin K1 on bone may not be mediated by BMD or bone turnover. Our findings are congruent with epidemiologic data from the Framingham Heart Study [24] and the Nurses' Health Study [5]. A recent meta-analysis of randomized controlled trials also showed that daily supplementation with vitamin K2 (45 mg of menaquinone 4) consistently reduces hip (odds ratio [OR] = 0.23, 95% CI 0.12 to 0.47), vertebral (OR = 0.40, 95% CI 0.25 to 0.65), and all nonvertebral fractures (OR = 0.19, 95% CI 0.11% to 0.35) [25]. In a recent study, Knapen and colleagues suggests that vitamin K2 supplementation improves bone strength via bone geometry rather than BMD [26]. Whether vitamin K1 affects bone geometry and bone quality rather than bone density, and whether there are differences in the effects of vitamins K1 and K2 on bone remains to be determined.

We also detected a lower incidence of cancers in the vitamin K–supplemented group. Recent data suggest that the K vitamins may have anticancer effects [27]. Most of the data have been on vitamin K3, especially as adjuvant therapy in conjunction with vitamin C in the treatment of hepatocellular cancer, leukemia, bladder, ovarian, prostate, and other cancers [28,29]. There are some data on the anticancer effects of vitamins K2 and K5 [30–34]. Our study may be the first to suggest that vitamin K1 also has anticancer effects. These effects may be mediated through tyrosine kinases [27].

Our study has several limitations. It was originally designed as a 2-y study with BMD as the primary outcome. Women were then recruited into a study extension of variable duration because follow-up visits were planned to terminate between March and September 2006. Thus, the number of women followed to 3 and 4 y was small, and the study was not powered to examine fracture outcomes. We used densitometric VFA for the assessment of morphometric vertebral fractures. VFA has been shown to be less precise than conventional lateral spine radiography in assessing for presence of morphometric vertebral fractures [35]. Our results on morphometric fractures could have been influenced by the use of this technique. In addition, the number of cancers observed was small, and a chance association cannot be ruled out. However, our observed fracture and cancer reduction is consistent with other research, reducing the likelihood that these are just chance findings.

In conclusion, vitamin K1 supplementation at a daily dose of 5 mg does not protect against age-related decreases in BMD, but may reduce the incidence of fractures and cancers in postmenopausal women with osteopenia. Additional studies need to be conducted to understand the potential mechanisms behind these observations. Before high-dose vitamin K1 supplementation can be recommended for general use, further randomized controlled trials have to be done to confirm these findings.

## Supporting Information

Text S1 ECKO Protocol 2001—Initial Protocol for the Trial (81 KB PDF)

Text S2 ECKO Extension Protocol 2005—Protocol for the Trial Extension (119 KB PDF)

Text S3 CONSORT Statement (58 KB DOC)

## Abbreviations

AE: adverse event
BMD: bone mineral density
CI: confidence interval
CTX: C-terminal telopeptide
CV: coefficient of variation
ECKO trial: Evaluation of the Clinical Use of Vitamin K Supplementation in Post-Menopausal Women With Osteopenia
Gla: gamma-carboxyglutamic acid
ICH: International Committee on Harmonization
OC: osteocalcin
OR: odds ratio
SAE: serious adverse event
SF-36: 36-item Short-Form General Health Survey
ucOC: undercarboxylated osteocalcin
UHN: University Health Network
VFA: vertebral fracture assessment
