# Correction: Vitamin K Supplementation in Postmenopausal Women with Osteopenia (ECKO Trial): A Randomized Controlled Trial

**DOI:** 10.1371/journal.pmed.0050247

**Published:** 2008-12-23

**Authors:** Angela M Cheung, Lianne Tile, Yuna Lee, George Tomlinson, Gillian Hawker, Judy Scher, Hanxian Hu, Reinhold Vieth, Lilian Thompson, Sophie Jamal, Robert Josse

Correction for:

Cheung AM, Tile L, Lee Y, Tomlinson G, Hawker G, et al. (2008) Vitamin K supplementation in postmenopausal women with osteopenia (ECKO Trial): A randomized controlled trial. PLoS Med 5(10): e196. doi:10.1371/journal.pmed.0050196

Three errors were made in the Funding and Acknowledgments sections of this article. In the Funding section, the “Wyeth Foundation” should be replaced by the “Centrum Foundation” and “Whitehall Robbins” should be spelled “Whitehall Robins.” In the Acknowledgments section, “Dr. Faten Al-Doori” should be spelled “Dr. Faten Al-Douri.”

