# Supplementary material for: Vitamin K Supplementation in Postmenopausal Women with Osteopenia (ECKO Trial): A Randomized Controlled Trial
Source: PLoS Med. 2008 Oct 14;5(10):1–12. doi: 10.1371/journal.pmed.0050196 (PMC2566998; doi:10.1371/journal.pmed.0050196)
Supplement: Supplementary file 1 [file pmed.0050196.sd001.pdf]

Cheung, Angela M University Health Network $152,563

Evaluation of the Clinical Use of Vitamin K Supplementation in Postmenopausal Women with Osteopenia

1.0 Literature Review

Osteoporosis and Osteopenia

Osteoporosis is major cause of morbidity and mortality in Canadian postmenopausal women. It is a systemic disease characterized by low bone mass and microarchitectural deterioration of bone tissue, resulting in bone fragility and an increased risk of fractures [1]. The loss of bone mass occurs when the rate of bone resorption is greater than that of bone formation, as in the case of postmenopausal osteoporosis. It is estimated that one in six women over the age of 50 has osteoporosis [2]. The lifetime risk of any osteoporotic fracture for an average 50 year-old Canadian woman is >40% [1]. Because osteoporosis often results in deterioration of functional status and quality of life, the burden it places on patients, their social supports, and the health care system is substantial.

Bone mineral density (BMD) is an independent predictor of fracture risk in postmenopausal women [3]. The risk of fractures increases with reduction in BMD, independent of age. The current standard is to express BMD in terms of T-scores, standard deviations below or above the young adult mean value for a selected standard population. In 1994, a group of experts convened by WHO established criteria for the definition of osteoporosis, based on bone mineral density [4]. Thus, osteoporosis is defined as T-score of –2.5 or below, normal is defined as T-score of –1 or above, and osteopenia is between –1 and –2.5. Women with BMD measurements in the osteopenic range have twice the risk for fragility fracture as compared to women in the normal range [3].

Risk for osteoporosis is greater for women than men. Established risk factors for women include: increased age, Caucasian or Asian ethnicity, postmenopausal status, late menarche or early menopause, low peak bone mass, family history of osteoporosis or fracture, low dietary intake of calcium and vitamin D, lack of physical activity, smoking, excess alcohol consumption, and long-term use of certain medications, such as steroids, anticonvulsants, immunosuppresants, and heparin [4;5]. Factors more recently receiving attention include vitamin K.

Vitamin K and Undercaroxylated Osteocalcin as a Marker for Vitamin K Status

There are two main types of vitamin K that occur naturally: vitamin K1 (phylloquinone), which is found in plants, and vitamin K2 (the menaquinones; MK), which are synthesized by bacteria in the gastrointestinal tract and found in meat, cheese and fermented products. The absolute and relative contribution of bacterial vitamin K to the pool used by the body is somewhat unclear [6;7].

There are several different forms of menaquinone (MK-1 to MK-14) [8], varying in the number of isoprenoid residues attached to the ring. All the vitamin K compounds possess a 2-methyl 1, 4-naphthoquinone ring. The functional group of the vitamin K is the naphthoquinone ring, so the mechanism of action is similar for all K vitamins [7].

Vitamin K is an essential cofactor for the carboxylation of glutamate to gamma-carboxyglutamic acid (Gla), which confers calcium-binding properties to vitamin K-dependent proteins. To date, two distinct groups of Gla-containing proteins have been identified: those involved in blood coagulation and those found in calcified tissue such as osteocalcin (bone Gla protein) and matrix Gla proteins. The role of Gla-containing proteins in the blood plasma is well understood; the function of Gla-containing proteins in calcified tissues is less clear.

The three currently known vitamin K-dependent bone proteins (osteocalcin, matrix Gla protein and protein S), are synthesized by osteoblasts. Osteocalcin (OC) is thought by some to be produced solely by bone tissue [7;9], but others have reported small amounts of OC in vascular smooth muscle cells [10]and in platelets [11]. OC is approximately 20% of the noncollagenous protein in bone [7;9]. Currently, the exact function of this protein is unclear. The carboxylated glutamic acid residues of OC facilitate calcium binding to hydroxyapatite [6], and are likely involved in bone mineralization [12;13]. However, some in vitro and knock-out gene studies suggest that OC may inhibit bone mineralization [14], and may act as a negative regulator for bone formation [9;15]. In general, OC is thought to be a marker of bone formation in postmenopausal states [7], and is likely to be both vitamin D and vitamin K dependent [9;15].

Undercarboxylated vitamin K-dependent proteins are nonfunctional [7;12]and their presence in serum has been used as a marker of Vitamin K status [7;9;15]. The carboxylation state of OC is responsive to changes in vitamin K intake levels [16-18]. While prothrombin time is a good indicator of vitamin K status for blood coagulation, it is not sensitive enough to identify deficiency states that would affect bone health. Thus, undercarboxylated osteocalcin (UcOC) is the preferred biochemical index for assessing vitamin K status with respect to bone health [19;20].

It is difficult to define “normal” or “abnormal” values for serum OC and/or UcOC, as the types of assays used in different laboratories vary [21;22]and results need to be standardized [22]. However, many studies have shown that UcOC concentrations increase with age, from about 20% of total OC in the third to fifth decade of life to 40-50% of total OC in the later years [16-19;23]. Increasing evidence seems to indicate that the present recommended requirements for vitamin K (1µg/kg body weight/day), which are based on blood coagulation times, may not be sufficient to ensure that all vitamin K-dependent proteins are in their maximally carboxylated forms [6 7;12].

Epidemiologic Data on Vitamin K and Bone Health

Recent data suggests a potential role for vitamin K in the bone health of postmenopausal women. Serum vitamin K concentrations appear to be strongly influenced by the polymorphism of apolipoprotein (Apo) E, which plays a role in vitamin K transportation in blood. Individuals with the E4 allele have been shown to have the lowest phylloquinone concentrations [24;25]. Apo E4 has also been associated with fracture risk and low BMD in studies of Caucasian and Japanese postmenopausal women [26-29].

The effect of long-term administration of oral anticoagulants (vitamin K antagonists) on bone is controversial. While a number of studies report a significant reduction in BMD [30-33], others have not detected a similar relationship. In a meta-analysis of nine cross-sectional studies, long-term exposure to oral anticoagulants was related to a significant decrease in BMD at the ultradistal radius, but not at the lumbar spine, femoral neck or femoral trochanter [34]. The Study of Osteoporotic Fractures examined bone density and fracture rates in 149 warfarin users compared to 6052 nonusers and did not find a difference between the two groups [35]. These studies have methodological issues that make interpretation and generalization of the results difficult: most studies are cross-sectional in nature; and the participants on anticoagulants often have comorbid conditions and poorer health.

A number of studies have also reported significantly lower circulating levels of vitamin K in fracture patients as compared to their age-matched counterparts [36-38]. Studies examining dietary intake of Vitamin K have reported an increased fracture risk among those in the lowest quintiles of dietary vitamin K1 intake as compared to those in the higher quintiles [39;40].

The Nurses' Health Study, a prospective cohort study of 72,327 women over a 10-year period, assessed the relationship between dietary vitamin K intake and fracture risk [39]. The median intake at baseline was 169 μg/day with a range of 41-604μg. Women in the lowest quintile (<109μg/day) experienced significantly higher fracture risk as compared to the third quintile (146-183μg/day). The difference between the remaining quintiles was not significant. The age-adjusted relative risk for hip fractures was 1.43 (95%CI: 1.08-1.89) for the lowest quintile as compared to the higher quintiles combined. A linear dose-response trend was not observed; however, a significant interaction between vitamin K and vitamin D was observed. Among women with low vitamin K intake (<109μg/day), those with high vitamin D intake (>8.4μg/day) had a greater fracture risk than those with low vitamin D intake (<4μg/day).

The only randomized placebo-controlled trials published to date have examined the relationship of vitamin K2 supplementation (45mg/day) to BMD or fracture incidence. All five studies were conducted in Japan [41-45]. The largest study followed a total of 205 osteoporotic women randomly assigned to receive either 45mg vitamin K2 (menatetrenone) plus calcium supplementation (150 mg elemental calcium/day) or calcium supplementation alone. Participants were prohibited from taking drugs that could affect bone and calcium metabolism. At the end of 2 years, the treated group experienced significantly less reduction in BMD (L2-L4) as compared to controls (-0.4$\pm$0.7% vs. -2.6$\pm$0.6%; p=0.019). As well, the incidence of clinical fractures was significantly lower in the treated group (13% vs. 30%; p=0.027). Serum levels of OC rose significantly in the treated group (42.4$\pm$6.9%) as compared to controls (18.2$\pm$6.1%), suggesting accelerated bone formation, while serum levels of cOC were significantly lower in the treated group (1.6$\pm$0.1 ng/ml vs. 3.0$\pm$0.3ng/ml; p<0.0001).

Of the remaining studies, one examined the effect of vitamin K2 supplementation among 72 postmenopausal women [42], one examined the effect among 110 women receiving leuprolide therapy [44], one examined the effect on 108 stroke patients [45], and another examined the effect among 20 men and premenopausal women with chronic glomerulonephritis [43]. These studies all observed similar suppression in bone loss among participants receiving vitamin K2 supplementation, though not all reached statistical significance. Of the two that measured markers of bone formation, only one found a significant difference in the treated group [44].

With respect to interventions with vitamin K1, there have been few published studies to date [16-18]. The largest study, a randomized placebo-controlled trial, involved 111 healthy women between the ages of 50 and 85 years randomized to receive either placebo or vitamin K1 at 1mg/day for 3 months [17]. Women were classified as fast or normal losers of calcuim based on calcuim/creatinine ratios in fasting urine samples. After one month of vitamin K supplementation, the urinary calcium loss was significantly less among the fast losers as compared to their matched controls and remained significant after the third month (mean change -30%; $p<0.005$). There was no effect seen among the normal calcium losers over the treatment period. Among women receiving vitamin K1, levels of serum cOC increased significantly, as did bone-specific alkaline phosphatase. Levels of UcOC remained unchanged.

A second study involved 20 postmenopausal osteoporotic women with previous Colles fractures [18]. The study used a cross-over design, randomly assigning women to initially receive either vitamin K1 (1mg) or a combination of vitamin K1 (1mg) plus vitamin D (400IU) for two weeks. After a 3 month 'washout' period, assignments were reversed. After two weeks of supplementation, total serum OC levels increased in both the vitamin K1 and vitamin K1+D groups but only reached significance in the latter group. Degree of carboxylation increased significantly in both groups ($p<0.001$). Carboxylation percentages returned to that observed at baseline 10 weeks after supplementation stopped. Urinary calcium, creatinine and bone-specific alkaline phosphatase (BAP) levels did not differ significantly before and after supplementation.

2.0 Rationale

Osteoporosis is a systemic disease that affects approximately one in six women over the age of 50 [2]. It is a condition characterized by low bone mass that is often undiagnosed until a fragility fracture is sustained, often resulting in chronic pain, disability, and decreased quality of life. Recent data from the Canadian Multicentre Osteoporosis Study (CaMOS) suggest that a further 45% of women over the age of 50 have osteopenia [2]. Pharmacological treatments have adverse effects, and are currently only recommended for use for the treatment of postmenopausal women with T-scores of –2 and below [46;47]. It is unclear whether osteopenic women with T-scores between –1 and –2 should be treated, even though their fracture risk is still greater than that of the women with normal bone mineral density.

Our review of the literature supports the role for vitamin K in bone health. Case-control studies have shown circulating levels of vitamin K1 and K2 to be significantly lower in patients with osteoporotic fractures as compared to healthy controls. Cohort studies have reported a relationship between dietary intake of vitamin K1 and fracture risk. The role of vitamin K in bone health is also supported by current clinical understanding of bone metabolism.

Randomized trials have shown a beneficial effect of vitamin K2 on BMD but vitamin K2 is not currently available in Canada. Smaller intervention trials using vitamin K1 have also shown promising results.

If vitamin K supplementation can be shown to reduce bone loss in postmenopausal women, it could provide a safe, non-pharmacological intervention for women at increased risk of fractures. In light of this, we propose to conduct a randomized placebo-controlled trial to determine if vitamin K supplementation administered over two years can prevent bone loss in postmenopausal women with osteopenia.

3.0 Research Objectives

Our primary objective is to examine whether vitamin K1 supplementation (5mg/day) affects spine (L1-L4) and total hip bone mineral density in postmenopausal women with osteopenia.

Our secondary objectives are to determine:

1. whether vitamin K1 supplementation affects levels of bone formation markers (serum OC, serum BAP) and bone resorption markers (serum N-telopeptide)
2. whether vitamin K supplementation affects degree of carboxylation of OC
3. the potential adverse effects from long-term vitamin K supplementation
4. whether vitamin K supplementation affects health-related quality of life (emotional and physical aspects)

Our primary hypothesis is that vitamin K supplementation prevents bone loss in postmenopausal osteopenic women. Our secondary hypotheses are: there will be an increase in markers of bone formation but no effect on markers of bone resorption; there will be an increase in the degree of carboxylation of OC; there will be no significant adverse effects associated with vitamin K supplementation at 5mg per day over a 2-year period; and there will be no change in health-related quality of life.

4.0 Methods

4.1 Study Design

We propose to carry out a single centre (University of Toronto) double-blind, placebo-controlled randomized trial. Approximately 400 postmenopausal women will be recruited using convenient sampling and randomly assigned to receive placebo or vitamin K supplementation for a period of two years. Since all women entering the study will have confirmed low bone mass, participants in both treatment groups will also receive calcium and vitamin D supplementation, if appropriate. Recruitment will take 1 year, and participants will be followed for 2 years from the date of randomization.

4.2 Study Population

The population of interest is postmenopausal women with low bone density, between T-scores of –1 and –2. Since rate of bone loss is greatest following menopause, we would expect to see the greatest benefit of a preventive intervention among postmenopausal women. Our study cohort will be restricted to women who are osteopenic rather than osteoporotic. This is because women with osteoporosis are at a significantly increased risk for fragility fractures, and there are current established clinical guidelines for treating postmenopausal women with a T-score below –2 [46;47].

Inclusion criteria: The following definitions will be used to determine eligibility for inclusion:

1. postmenopausal
   - one year since the natural cessation of menses, or
   - hysterectomy with either postmenopausal status confirmed by FSH lab values, or age 55 and above

2. osteopenic
   - T-score between -1 and -2 on lumbar, total hip or femoral neck BMD measurement (the lowest reading of the above three measurements must be above -2)
   - based on documented BMD done within the past 6 months or BMD measurement done at screening

Exclusion criteria: The following exclusion criteria will be applied to ensure safety of the participants and to enhance internal validity of the study: women ever having had a fragility fracture; women on anticoagulants in the past 3 months; women on hormone replacement therapy, bisphosphonates, raloxifene, or calcitonin during the past 3 months; women diagnosed with Paget's disease, hyperparathyroidism, or hyperthyroidism; women with decompensated liver disease, kidney disease, pancreatic disease, lung disease, or heart disease; women taking mega-doses of vitamin A or E; women with a history of cancer in the past 5 years; women involved in other clinical trials; and any women who, in the opinion of the principal investigator, is at poor medical or psychiatric risk for the study.

Recruitment: We will recruit a convenience sample of 400 women through family practices, osteoporosis clinics, community outreach, and media promotion. Our group has successfully recruited women for several studies, including randomized trials, using this method.

4.3 Sample Size/Power Calculations

Our sample size calculations are based on our primary outcome, which is the difference in the changes in spine (L1-L4) and total hip BMD (delta) between the vitamin K group and the placebo group at the end of 2 years, expressed as g/cm^2. Assuming equal between-subject variances at each measurement point, the variance for delta (the difference between the two groups) can be calculated using the following formula:

Var (delta) = Var + Var –2 rho Var

where Var is the variance that include between subject and within subject variance, and rho is the correlation coefficient of BMD over time. As there is no published data on the correlation coefficient for BMD, we assume rho to be in the range of 0.5 to 0.8.

Below is a table showing the various sample sizes for the expected differences between the two groups (deltas), using a significance level of 95% (alpha = 0.05) and a power of 80% (1-beta = 0.8):

Number of subjects in each group	Rho	Expected Delta in Spine (g/cm^2)	Expected Delta in Total Hip (g/cm^2)
100	0.5	0.0437	0.0490
	0.6	0.0391	0.0438
	0.7	0.0339	0.0379
	0.8	0.0276	0.0310
150	0.5	0.0357	0.0400
	0.6	0.0319	0.0358
	0.7	0.0276	0.0310
	0.8	0.0226	0.0253
200	0.5	0.0309	0.0346
	0.6	0.0276	0.0310
	0.7	0.0239	0.0268
	0.8	0.0195	0.0219
300	0.5	0.0252	0.0283
	0.6	0.0226	0.0253
	0.7	0.0195	0.0228
	0.8	0.0160	0.0179

Based on an assumption of 10% withdrawal rate (stopping study medication) per year and a 5% drop out rate (lost to followup) per year, we estimate that we will need to recruit 200 patients per group (total of 400 patients) in order to observe a clinically meaningful and statistically significant difference between the two groups.

4.4 Intervention

Exposure definition: Women assigned to the treatment group will receive 5mg of vitamin K1 per day in the form of capsules. Women assigned to the placebo group will receive identical capsules containing the same solution without vitamin K1. Following assessment of dietary intakes, women in both groups may receive calcium and vitamin D supplementation so that daily intakes will approximate recommended levels (1500mg Ca, 800-1000iu Vitamin D).

We are confident that Vitamin K1 at 5mg per day is adequate to observe a therapeutic effect and can be safely administered. Currently, doses of 5 to 10mg of vitamin K1 are being administered safely as short term treatment for bleeding disorders. As well, unpublished randomized studies in the Netherlands have administered doses of vitamin K1 ranging from 1-10mg for up to five years and have observed no significant adverse events (C. Vermeer, personal communication, February 2001).

Randomization: Participants will be assigned to treatment groups using a randomly generated list, which will not be accessible to the investigators or study personnel. The Toronto General Research Institute Clinical Resource Center will work with the pharmacy in generating the list. Block randomization will be used to ensure that there are equal numbers of participants assigned to each treatment group at the end of the study.

Blinding: In order to minimize bias, the study will be double-blinded so that neither the participants nor the investigators or study staff will be aware of the participant assignments. It is unlikely that assignments will be discerned from patient information and unanalysed serum samples obtained by study staff during follow-up visits.

Patients should not be able to discern whether they are receiving vitamin K or placebo since the appearance will be identical and there should be no taste associated with the capsules. We expect adverse events due to vitamin K to be minimal and unlikely to conclusively reveal patient assignment.

It is possible that changes in BMD measurement could lead to changes in behaviour on the part of the participant or attending staff and may introduce bias. To prevent this, BMD measurements made annually will not be available to participants or study staff until the end of the study period. Results will, however, be reviewed by two co-applicants who will not have direct participant contact (Drs. Hawker and Josse). Any significant changes which would necessitate a change in care will be communicated to the study staff and reported to the safety committee. Participants whose T-scores fall below -2.5 (ie. in the osteoporotic range) at 1 year will be withdrawn from the study and counselled with respect to treatment options for osteoporosis.

Results of blood samples can also reveal patient assignment, particularly since these analyses will involve determining circulating levels of K1 and K2. Results will be retained in the laboartory and reviewed for quality control by two co-applicants with no direct participant contact (Drs. Vieth and Thompson).

Drug Administration:

Participants will receive supplies of 6 months' worth of capsules (~180) at the baseline visit and at each semiannual visit thereafter and will be instructed as to how and when to take them. At each follow-up visit, participants will be asked to bring with them any unused capsules. These will be counted and recorded as an indicator of compliance.

4.5 Outcome Definition

The primary outcome of interest is change in BMD. Secondary outcomes of interest are biochemical markers of bone turnover and bone resorption, degree of carboxylation of OC, adverse effects, and quality of life.

BMD measurements: Bone mineral density in the lumbar spine (L1-L4) and left total hip will be measured by Dual Energy X-ray Absortiometry (DEXA). An experienced technologist will use standardized methods to acquire and analyze scans using Hologic QDR 4500A (Hologic Inc,

Waltham, Massachusetts) at the University Health Network Osteoporosis Program Bone Density and Body Composition Laboratory. Recent precision studies were performed in this laboratory using the method described by Bonnick et al. [3]. Thirty-two volunteers with T-scores ranging from –2.9 to 4.1 were scanned twice with on-and-off the scanning table and repositioning in between scans. Our results show coefficient of variations of 0.89 % for the lumbar spine (L1-L4) and 1.09% for the total hip, which are consistent with other published data.

Daily and quarterly quality control methods are performed in this laboratory. The quality control logs over the study period will be reviewed as part of quality assessment of the bone density scans in the study.

Biochemical markers: Serum OC will be measured using a radioimmunoassay kit (Incstar, Stillwater, Minnesota) as described by Knapen et al [16]. Serum UcOC, as a measure of vitamin K status, will be measured based on different affinities of cOC and UcOC for hydroxyapatite. Briefly, 300μL samples will be incubated with 30 mg hydroxyapaptite (calcium phosphate tribasic type IV, Sigma Chemical Co) in Eppendorf tubes, mixed end-over-end for 1 hour at 4°C and then centrifuged. The supernatant will be decanted. The unbound OC remaining in the serum supernatant will be measured as above, with radioimmunoassay, and the amount of UcOC will be calculated by subtracting unbound OC from total OC. UcOC level will be expressed as a percentage of total OC concentration. The limit of detection for OC is 0.78 ug/L.

Serum levels of cross-linked N-telopeptides of type I collagen (NTx) will be measured using a microplate enzyme-linked immunosorbent assay (ELISA) in a competitive inhibition format as described by Scariano et al. [48]. This assay uses a specific monoclonal antibody (MAb1H11). Results will be reported as nanomoles of bone collagen equivalents (BCE)/L. The limit of detection is 1.0 nmol BCE/L.

Serum bone specific alkaline phosphatase (BAP) will be measured using a commercially available immunoassay kit in a microtiter strip format with a monoclonal anti-BAP antibody coated to the strips (Alkphase-B, Metra Biosystems, Mountain View, CA).

Adverse Events: The only side-effect associated with vitamin K1 supplementation that we are aware of is nausea. Participants will be asked about any adverse events at each follow-up visit and will be asked to contact the study office should any conditions arise between visits. Participants will be asked to report any untoward medical occurrence occurring over the study period, without regard to causal relationship. Data will be captured on a standardized reporting form (see appendix A) and all events will be reported to the safety committee.

Health-Related Quality of life:

Two instruments will be used to measure changes in quality of life: the Osteoporosis Quality of Life Questionnaire (OQLQ) [49]and the Medical Outcomes Survey 36-item short form questionnaire (SF-36) [50;51]. Both have been tested for reliability and validity.

The OQLQ is a 30-item questionnaire which measures each of the following domains on a seven-point scale: symptoms, emotional function, physical function, activities of daily living, and leisure activities. This questionnaire is specific to postmenopausal women with back pain

resulting from osteoporosis. Because women in our study are osteopenic and likely to be asymptomatic, we will also administer the SF-36, which assesses nine health domains: physical functioning, role functioning-physical, bodily pain, general health, vitality, social functioning, role functioning- emotional, mental health and reported health transition. Among postmenopausal osteoporotic women, the SF-36 was found to be more powerful than the OQLQ in assessing emotional function. The SF-36 is self-administered and can be completed in less than 15 minutes [51;52]

4.6 Data Collection

Participants will be followed for a total of two years from randomization (baseline). Follow-up visits will occur at 3, 6, 12, 18, 24 months after the baseline visit.

In addition to the outcome measures, we will collect data which will allow us to do the following:

1. verify the comparability of treatment groups, eg. patient demographics, health behaviors, medical history, diet, etc.
2. help assess the generalizability of the study cohort, eg. age, ethnicity, socio-economic status, etc.
3. ensure the health of our participants is not being compromised
4. monitor changes in circulating vitamin K levels

Collection of demographic data, medical history, and dietary intake: The data collection instruments will be administered by study staff during follow-up visits as shown in appendix A. Data to be collected include: patient demographics, lifestyle, patient medical history, medication use, ongoing medical conditions, and dietary and physical activity assessment.

Collection and analysis of serum samples: Fasting blood samples will be collected from participants at baseline, 3, 6, 12, and 24 months. Blood will be collected by venipuncture, centrifuged, and the plasma portion stored.

Serum vitamin K1 and K2 levels will be measured by gas chromatography / mass spectroscopy using Agilent 5973 Network Mass Selective Detector interfaced with Agilent 6890 Series Gras Chromatograph. The protocol will be based on the method of Fauler et al [53]. Internal standards will be purchased from Sigma Chemical Co.

Serum Calcium, Magnesium, Phosphate, Creatinine, Albumin, vitamin D Levels will also be measured using standard protocols.

4.7 Steering Committee and Quality Control

A Steering Committee consisting of all co-applicants will be established to: review issues around recruitment and retention; monitor data quality via random chart reviews; develop consensus on arising issues; and advise the principal investigator on study related issues. The committee will meet quarterly for the first year and a half, and semi-annually for the rest of the study. Drs.

Adachi and Papaioannou from the Safety Committee will attend the steering committee meetings on an annual basis.

4.8 Data Analysis

For all analyses, we will analyse data on the principle of intent to treat. Significance will be assessed at a level of $\alpha=0.05$.

Baseline characteristics for each group will be summarized and tested for statistically significant differences using the Chi-square p-values for categorical data and 2 sample t-test for continuous variables. As well, secondary diagnoses not related to the study and medication use will be summarized for each treatment group at baseline and at 24 months.

In the case of withdrawals, we will ask that they return at the two year mark for final BMD and other measurements; or, if not possible or inappropriate, we will attempt to get final BMD measurement and serum samples at the time of withdrawal. We will summarize patient characteristics and reasons for withdrawals by treatment group. We will also determine whether participants who withdraw from the study differ from those who remain in terms of factors relating to the primary outcome of interest.

Analysis of the primary outcome: The primary objective of this study is to determine whether vitamin K supplementation affects BMD. The outcome for this analysis will be the changes in spine (L1-L4) and total hip BMD measurement at baseline and at two years of follow-up in the two groups. This will be assessed for significance using a 2 sample t-test.

Analyses of secondary outcomes: Changes in bone formation markers and degree of carboxylation of osteocalcin will be analysed comparing differences in means and standard deviations between groups, at baseline and at 24 months, and changes within groups. One-way analysis of variance (ANOVA) will be used to compare treatment groups. In addition, the differences between the two groups will be assessed by the 2-tailed Mann-Whitney U test.

Analysis of adverse events will consider both number and type of adverse events in each group, and will be performed using the Chi-square test and the Fischer Exact Test. Mean changes in quality of life measures will be analysed for each domain separately, using two sample t-test.

5.0 Safety

An arms-length safety committee will monitor safety issues throughout the study. Dr. Jonathan Adachi and Dr. Alexandra Papanniouau of McMaster University (Hamilton) will serve on this committee. All adverse events and significant medical conditions will be recorded and faxed to the committee for adjudication, when reported by the participant.

Patients will be discontinued from the study if they develop clinical fractures, if their BMD falls below –2.5 at 1-year, or if they develop medical conditions that necessitate going off vitamin K (for example, deep vein thrombosis and pulmonary embolism).

6.0 Personnel

Dr. Cheung, the principal applicant, is an Assistant Professor in the Departments of Medicine, Public Health Sciences, Health Administration and the Clinical Epidemiology and Health Care Research Program at the Univerity of Toronto. She is the Director of the Osteoporosis Program and the Associate Director of the Women's Health Program at the University Health Network. She is currently holding a 5-year Ontario Ministry of Health Health Services Research Career Scientist Award. She was a member of the Ontario Women's Health Council Strategic Working Group on Osteoporosis, and recently published a framework and strategy for the prevention and management of osteoporosis. She is also working with the Canadian Task Force for Preventive Health Care and the Osteoporosis Society of Canada on the revised Canadian guidelines for osteoporosis in postmenopausal women. She has been successful in conducting several randomized controlled trials in postmenopausal women – the 10,000-women RUTH (Raloxifene Use for the Heart) trial and the 22,000-women STAR (Study of Tamoxifen and Raloxifene for the prevention of breast cancer) trial. The Toronto site for the RUTH trial is the coordinating centre for Canada. Dr. Cheung will be responsible for the overall design, conduct and analysis of the study, and the preparation of the manuscript.

Dr. Hawker (Associate Professor in Medicine) and Dr. Josse (Professor in Medicine) are Directors of Osteoporosis Programs at Sunnybrook and Women's Health Sciences Centre and St. Michael's Hospital respectively. Dr. Hawker is also the Director for the Clinical Epidemiology and Health Care Research Program at University of Toronto. Both Drs. Hawker and Josse have held multiple grants on osteoporosis. Dr. Hawker is currently holding a 5-year CIHR Career Scientist Award. Both Drs. Hawker and Josse will be responsible for reviewing the one-year BMD scans to see if the change in BMD warrants withdrawal from the study. Dr. Murray (Professor in Medicine) is an expert in the area of metabolic bone disease and will provide content and methodological expertise in the design and conduct of the study. Dr. Lee (Instructor in Medicine) is an expert in postmenopausal osteoporosis. She will assist in patient assessments in the study. Dr. Patricia Massicotte (Assistant Professor in Medicine and Pediatrics) is a hematologist and an expert in the link between coagulation and bone disease. She will provide content and methodological expertise in the design and conduct of the study.

Dr. Reinhold Vieth (Associate Professor in Laboratory Medicine and Pathobiology) is the Director and Clinical Biochemist of the Bone and Mineral Laboratory at Mount Sinai Hospital. He has an active CIHR grant examining Vitamin D supplementation in postmenopausal women. He will be overseeing the biochemical measurements of bone turnover markers, vitamin D and calcium. Dr. Lillian Thompson (Professor in Nutrition Sciences and Medicine) is an expert in the area of nutrition and bone health in postmenopausal women and will oversee the food record data collection and analysis.

Dr. Jonathan Adachi (Professor in Medicine at McMaster University) and Dr. Alexandra Papaiannou (Assistant Professor in Medicine at McMaster University), both osteoporosis researchers, will serve on our Safety Committee, and will provide arm's length monitoring of adverse events for our study.

7.0 Timeline

This is expect to be a four year study. The first one and half years will be required for recruitment of participants, the subsequent two years will be required to complete follow-up, and an additional six months will be required for data cleaning, data analysis and manuscript preparation.

8.0 Confidentiality and Ethics

All participant data will be kept strictly confidential. All completed forms and test results will be kept in a locked cabinet in the study office. Participants will not be identifiable from any results published or presented as a result of this study.

All participants will be required to read an information sheet and sign a consent form before being enrolled in the study (appendix B). All potential participants identified as osteoporotic or osteopenic, whether or not they agree to participate, will be offered counselling with respect to osteoporosis and a clinical note will be provide for their primary care physician.

The study protocol is currently under ethics review at the University Health Network (see attached letter).

9.0 Relevance

This University of Toronto study will be the first randomized double-blind controlled trial examining the effect of vitamin K supplementation in postmenopausal osteopenic women in Canada. It will also be the largest performed to date in this population internationally. The results of this study can change our current concept of vitamin K requirements for optimal bone health. It will also inform us as to the potential adverse effects of long-term vitamin K supplementation. Since osteopenia affects 45% of women age 50 and over in Canada, the results of this study can have a significant impact in the care of these women, and in the field of osteoporosis.

List of Appendices

Appendix A. Data collection schedule and instruments
Appendix B. Patient consent form and information brochure
Appendix 1. Letters of collaboration and support

Reference List

1. Consensus development conference. Diagnosis, prophylaxis, and treatment of osteopororis. Am J Med 94, 646-650. 1993.

2. Tenenhouse A, Joseph L Kreiger N Poliquin S Murray TM Blondeau L Berger C Hanley CA Prior JC and the CaMos Research Group. Estimation of the prevalence of low bone density in Canadian women and men using a population-specific DXA reference standard: the Canadian multicentre osteoporosis study (CaMos). Osteoporos Int 11, 897-904. 2000.

3. Bonnick SL. Bone Densitometry in Clinical Practice. New Jersey: Humana Press Inc., 1998.

4. Genant, H. K., Cooper, C., Poor, G., and et al. Interim report and recommendations of the World Health Organization Task Force for Osteoporosis. Osteoporos Int 10, 259-264. 1999.

5. Cummings, S. R., Nevitt, M. C., Browner, W. S., and et al. Risk factors for hip fracture in white women. N Engl J Med 332, 767-773. 1995.

6. Shearer MJ. Vitamin K. The Lancet 345, 229-234. 1995.

7. Vermeer C, Schurgers LJ. A Comprehensive Review of Vitamin K and Vitamin K Antagonists. Hematology/Oncology Clinics of North America 14, 339-352. 2000.

8. New SA. Bone health: the role of micronutrients. Br.Med.Bull 55, 619-33. 1999.

9. Ferland G. The Vitamin K-dependent Proteins: An Update. Nutr Rev 56, 223-230. 1998.

10. Severson AR, Ingram RT Fitzpatrick LA. Matrix proteins associated with bone calcification are present in human vascular smooth muscle cells grown in vitro. In Vitro Cell Dev Biol Anim 31, 853-857. 1995.

11. Thiede MA, Smock SL Petersen DN Grasser WA Thompson DD Nishimoto SK. Presence of messenger ribonucleic acid encoding osteocalcin, a marker of bone turnover, in bone marrow megakaryocytes and peripheral blood platelets. Endocrinology 135, 929-937. 1994.

12. Kohlmeier M, Salomon A, Saupe J, Shearer MJ. Transport of vitamin K to bone in humans. J.Nutr 126, 1192S-6S. 1996.

13. Weber P. Management of osteoporosis: is there a role for vitamin K? Int.J.Vitam.Nutr.Res 67, 350-6. 1997.

14. Price PA. Role of vitamin-K dependent proteins in bone metabolism. Ann Rev Nutr 8, 565-583. 1988.

15. Booth SL. Skeletal functions of vitamin K-dependent proteins: not just for clotting anymore. Nutr Rev 55, 282-284. 1997.

16. Knapen MH, Hamulyak K, Vermeer C. The effect of vitamin K supplementation on circulating osteocalcin (bone Gla protein) and urinary calcium excretion. Ann.Intern.Med 111, 1001-5. 1989.

17. Knapen MH, Jie KS, Hamulyak K, Vermeer C. Vitamin K-induced changes in markers for osteoblast activity and urinary calcium loss. Calcif.Tissue Int 53, 81-5. 1993.

18. Douglas AS, Robins SP, Hutchison JD, Porter RW, Stewart A, Reid DM. Carboxylation of osteocalcin in post-menopausal osteoporotic women following vitamin K and D supplementation. Bone 17, 15-20. 1995.

19. LJ Sokoll, JA Sadowski. Comparison of biochemical indexes for assessing vitamin K nutritional status in a healthy adult population. Am J Clin Nutr 63, 566-573. 1996.

20. Rucker R. Improved functional endpoints for use in vitamin K assessment: important implications for bone disease. Am J Clin Nutr 65, 883-884. 1997.

21. Binkley NC,.Suttie JW. Vitamin K nutrition and osteoporosis. J.Nutr 125, 1812-21. 1995.

22. CM Gundberg, SD Nieman S Abrams H Rosen. Vitamin K Status and Bone Health: An Analysis of Methods for Determination of Undercarboxylated Osteocalcin. J Clin Endocrinol Metab 83, 3258. 1998.

23. P Szulc, M.-C Chapuy PJ Meunier PD Delmas. Serum Undercarboxylated Osteocalcin Is a Marker of the Risk of Hip Fracture in Elderly Women. J Clin Invest 91, 1769-1774. 1993.

24. Saupe, J., Shearer, M. J., and Kohlmeier, M. Phylloquinone transport and its influence on -caroxyglutamate residues of osteocalcin in patients on maintenance homodialysis. Am J Clin Nutr 58, 204-208. 1993.

25. Kohlmeier M, Saupe, J., Drossel, H. J., and Shearer, M. J. Variation in phylloquinone (vitamin K1) concentrations in hemodialysis patients. Thromb Haemost 74, 1252-1254. 1995.

26. Salamone, L. M., Cauley, J. A., Zmuda, J., Pasagian-Macaulay, A., and et al. Apolipoprotein E gene polymorphism and bone loss: estorgen status modifies the influence of apolipoprotein E on bone loss. J Bone Miner Res 15, 308-314. 2000.

27. Johnston JM, Cauley JA, Ganguli M. APOE 4 and hip fracture risk in a community-based study of older adults. J.Am.Geriatr.Soc 47, 1342-5. 1999.

28. Cauley JA, Zmuda JM, Yaffe K, Kuller LH, Ferrell RE, Wisniewski SR et al. Apolipoprotein E polymorphism: A new genetic marker of hip fracture risk--The Study of Osteoporotic Fractures. J.Bone Miner.Res 14, 1175-81. 1999.

29. Shiraki, M., Shiraki, Y., Aoki, C., Hosoi, T., and et al. Association of bone mineral density with apolipoprotein E phenotype. J Bone Miner Res 12, 1438-1445. 1997.

30. Philip WJ, Martin JC, Richardson JM, Reid DM, Webster J, Douglas AS. Decreased axial and peripheral bone density in patients taking long- term warfarin. QJM 88, 635-40. 1995.

31. Monreal M, Olive A Lafoz E Del Rio L. Heparins, coumarin, and bone density. Lancet 338, 706. 1991.

32. Resch H, Pietschmann P Krexner E Willvonseder R. Decreased peripheral bone mineral content in patients under anticoagulant therapy with phenprocoumon. Eur.Heart J 12, 439-441. 1991.

33. Fiore CE, Tamburino C, Foti R, et al. Reduced axial bone mineral content in patients taking oral anticoagulant. South Med J 85, 538-42. 1990.

34. Caraballo PJ, Heit JA, Atkinson EJ, Silverstein MD, O'Fallon WM, Castro MR *et al*. Long-term use of oral anticoagulants and the risk of fracture. Arch.Intern.Med 159, 1750-6. 1999.

35. Jamal SA, Browner WS, Bauer DC, Cummings SR. Warfarin use and risk for osteoporosis in elderly women. Study of Osteoporotic Fractures Research Group. Ann.Intern.Med 128, 829-32. 1998.

36. Hodges SJ, Akesson K, Vergnaud P, Obrant K, Delmas PD. Circulating levels of vitamins K1 and K2 decreased in elderly women with hip fracture. J.Bone Miner.Res 8, 1241-5. 1993.

37. Hodges SJ, Pilkington MJ, Stamp TC, Catterall A, Shearer MJ, Bitensky L *et al*. Depressed levels of circulating menaquinones in patients with osteoporotic fractures of the spine and femoral neck. Bone 12, 387-9. 1991.

38. Bitensky L, Hart JP, Catterall A, Hodges SJ, Pilkington MJ, Chayen J. Circulating vitamin K levels in patients with fractures. J.Bone Joint Surg.Br 70, 663-4. 1988.

39. Feskanich D, Weber P, Willett WC, Rockett H, Booth SL, Colditz GA. Vitamin K intake and hip fractures in women: a prospective study. Am.J.Clin.Nutr.69, 74-9. 1999.

40. Booth SL,.Mayer J. Warfarin use and fracture risk. Nutr.Rev 58, 20-2. 2000.

41. Shiraki M.Shiraki Y.Aoki C.Miura M. Plasma Concentrations after Itravenous Administration of Phylloquinone (Vitamin K1) in Preterm and Sick Neonates. Thrombosis Research 99, 467-472. 2000.

42. Iwamoto I, Kosha S, Noguchi S, Murakami M, Fujino T, Douchi T *et al*. A longitudinal study of the effect of vitamin K2 on bone mineral density in postmenopausal women a comparative study with vitamin D3 and estrogen-progestin therapy. Maturitas 31, 161-4. 1999.

43. Yonemura K, Kimura M, Miyaji T, Hishida A. Short-term effect of vitamin K administration on prednisolone-induced loss of bone mineral density in patients with chronic glomerulonephritis. Calcif.Tissue Int. 66, 123-8. 2000.

44. Somekawa, Y., Chigughi, M., Harada, M., and Ishibashi, T. Use of vitamin K_2 (Menatetrenone) and 1,25-dihydroxyvitamin D_3 in the preventionof bone loss induced by Leuprolide. J.Clin.Endocrinol.Metab 84, 2700-2704. 1999.

45. Sato, Y., Honda, Y., Kuno, H., and Oizumi, K. Menatetrenone ameliorates osteopenia in disuse-affected limbs of vitamin D- and K-deficient stroke patients. J Bone Miner Res 23, 291-296. 1998.

46. Council of the National Osteoporosis Foundation. Guidelines for the early detection of osteoporosis and prediction of fracture risk. S Afr Med J 86, 1113-1116. 1996.

47. Scientific Advisory Board, Osteoporosis Society of Canada. Clinical Practice Guidelines for the Diagnosis and Management of Osteoporosis. CMAJ 155, 1113-1132. 1996.

48. Scariano JK, Glew RH Bou-Serhall CE Clemens JD Garry PJ Baumgartner RN. Serum levels of cross-linked N-telopeptides and aminoterminal propeptides of Type I collagen indicate low bone mineral density in elderly women. J Bone Miner Res 23, 471-477. 1998.

49. Osteoporosis Quality of Life Study Group. Measuring quality of life in women with osteoporosis. Osteoporos Int 7, 478-487. 1997.

50. Ware, J. E. and Sherbourne, C. D. The MOS 36-item Short-Form Health Survey (SF-36). I. Conceptual framework and item selection. Med Care 30, 437. 1992.

51. McHorney, C. A., Ware, J. E., and Raczek, A. E. The MOS 36-item Short-Form Health Survey (SF-36). II. Psychometric and clinical tests of validity in measuring physical and mental health constructs. Med Care 31, 247-263. 1993.

52. McHorney CA, Ware JE Rogers W et al. The validity and relative precision of MOS short- and long-form health status scales and Dartmouth COOP charts: results from the Medical Outcomes Study. Med Care 30, MS253. 1992.

53. Fauler G, Leis HJ Schalamon J Muntean W Gleispach H. Method for the Determination of Vitamin K1(20) in Human Plasma by Staple Isotope Dilution/Gas Chromatography/Mass Spectrometry. J Mass Spectro 31, 655-660. 1996.
