# Supplementary material for: Vitamin K Supplementation in Postmenopausal Women with Osteopenia (ECKO Trial): A Randomized Controlled Trial
Source: PLoS Med. 2008 Oct 14;5(10):1–12. doi: 10.1371/journal.pmed.0050196 (PMC2566998; doi:10.1371/journal.pmed.0050196)
Supplement: Supplementary file 2 [file pmed.0050196.sd002.pdf]

Research Protocol: CIHR Renewal 2004

Evaluation of the Clinical use of Vitamin K Supplementation in Postmenopausal Women with Osteopenia (the ECKO Trial)

Principal Investigator:

Dr. Angela Cheung, Director, Osteoporosis Program, University Health Network

0. THE NEED FOR A RENEWAL GRANT

In 2001, CIHR funded our research team to conduct a 2-year randomized control trial titled "**E**valuation of the **C**linical use of vitamin **K** supplementation in postmenopausal women with **O**steopenia" (the ECKO trial). Our original proposal was funded for 4 years (recruitment was estimated to take 1.5 years, trial duration was estimated to take 2 years, and initial application to Health Canada for an investigational drug number and final data cleaning and analyses was estimated to take 0.5 years).

We are currently applying for a 1.75-year renewal grant to:

1) Complete the originally proposed trial – our recruitment has taken 3 years instead of 1.5 years to complete.
2) Expand our evaluated outcomes to include assessment of fragility fractures, as suggested by our CIHR reviewers, and determination of whether apolipoprotein (Apo) E gene polymorphism modulates the effect of vitamin K on bone.

As well, we are including minor amendments to our original protocol (bolded in this research proposal) and details of the ECKO trial extension (Appendix 3), which does not significantly impact on this renewal budget.

1. THE NEED FOR A TRIAL

1.1 What is the problem to be addressed?

Osteoporosis is a systemic disease that affects approximately one in six women over the age of 50 in Canada [1]. It is a condition characterized by low bone mass that often goes undiagnosed until a fragility fracture occurs, often resulting in chronic pain, disability, and decreased quality of life. Recent data from the Canadian Multicentre Osteoporosis Study (CaMOS) suggest that a further 45% of women over the age of 50 have osteopenia [1]. Osteopenia is defined by the World Health Organization (WHO) in postmenopausal women as a bone mineral density (BMD) T-score between -1 to -2.5 as measured by Dual-Energy X-ray Absorptiometry; osteoporosis is defined as a T-score of -2.5 and below, or having had a fragility fracture[2]. While data from randomized controlled trials support the use of pharmacological therapies for the treatment of postmenopausal osteoporosis to prevent fractures, these therapies are generally not recommended for use in healthy postmenopausal women with osteopenia, especially when T-scores are above -2.0[3, 4], or above -1.5 with no additional risk factors[5] .

Current medical literature suggests that vitamin K plays an important role in bone metabolism. Case-control studies have shown circulating levels of vitamin K1 and K2 to be significantly lower in patients with osteoporotic fractures as compared to healthy controls[6-8]. Cohort studies have reported a relationship between dietary intake of vitamin K1 and fracture risk[9,10]. Randomized trials have shown a beneficial effect of vitamin K2 on BMD, but vitamin K2 is not currently available in Canada. Smaller intervention trials using vitamin K1 have also shown promising results.

If vitamin K1 supplementation can be shown to reduce bone loss in postmenopausal women, it could provide a safe, non-pharmacological intervention for women at increased risk of fractures. In light of this, we have been conducting a randomized placebo-controlled trial to determine if vitamin K1 supplementation administered over two years can prevent bone loss in postmenopausal women with osteopenia.

1.2 What are the principal research questions to be addressed?

Our primary objective is to examine whether vitamin K1 supplementation (5mg/day) prevents bone loss at the spine (L1-L4) and the total hip as measured by BMD in postmenopausal women with osteopenia. Our secondary objectives are to determine:

1. The potential adverse effects from long-term vitamin K1 supplementation,
2. Whether vitamin K1 supplementation affects levels of bone formation markers (serum osteocalcin (OC) and serum bone-specific alkaline phosphatase (BAP)) and bone resorption markers (serum N-telopeptide (NTx)),
3. Whether vitamin K1 supplementation affects the degree of carboxylation of OC, a major vitamin K-dependent protein in bone,
4. Whether vitamin K1 supplementation affects health-related quality of life,
5. **Whether vitamin K1 supplementation decreases the risk of having fragility fractures,**
6. **Whether ApoE modulates the effect of vitamin K on bone.**

Our primary hypothesis is that vitamin K1 supplementation prevents bone loss in postmenopausal osteopenic women. Our secondary hypotheses are: vitamin K supplementation at 5mg per day will have no significant adverse effects over a 2-year period; will increase bone formation markers but has no effect on bone resorption markers; will increase the degree of carboxylation of OC; will not affect measures of health-related quality of life; and will decrease the risk of fragility fractures.

There has been a high level of international interest in our study. This study will be the first to examine the long-term safety of vitamin K1 supplementation at 5mg per day. Our research group decided to extend the study for another 1-2 years. This will provide more long term safety data as well as fracture data. It will incur minimum additional costs as it will take us 2 more years to complete the trial, until end of August 2006. Our ECKO extension will involve recruiting those participants who have already completed their 2 years to continue for another 1 to 2 years, until August 2006. We have already received ethics approval and approval from Roche for the supply of vitamin K.

1.3 Why is a trial needed now?

The necessity for conducting this trial now is precipitated by the recent data supporting the role of vitamin K in bone health, as well as the fact that osteopenia and osteoporosis are becoming important public health issues globally. There are more than 4 million postmenopausal women in Canada; approximately one in two has osteopenia and another one in six has osteoporosis[1]. Epidemiologic observational studies have shown that low vitamin K intake is associated with low BMD[11-16] and increased osteoporotic fracture risk in postmenopausal women[9,10]. There have been several interventional studies, mainly examining the effect of vitamin K2 supplementation. The few clinical trials that have investigated vitamin K1 supplementation in postmenopausal women either did not have BMD or fractures as outcomes or had methodological issues limiting the validity and generalizability of the results. If vitamin K1 supplementation can be shown to reduce bone loss in postmenopausal women, it could provide a safe, non-pharmacological intervention for women at increased risk of fractures.

1.4 Give references to any relevant systematic review(s) and discuss the need for your trial.
There have been a few reviews on the role of vitamin K in bone health over the past few years[17-21]. None of them are systematic reviews. In the following section, we will discuss the various forms of vitamin K and their differences, the effect of vitamin K on bone proteins, such as osteocalcin, and the epidemiologic data supporting the hypothesis that vitamin K supplementation may be beneficial for bone health.

Vitamin K and Undercarboxylated Osteocalcin as a Marker for Vitamin K Status
There are two main types of vitamin K that occur naturally: vitamin K1 (phylloquinone), which is found in plants, and vitamin K2 (the menaquinones; MK), which are synthesized by bacteria in the gastrointestinal tract and found in meat, cheese and fermented products. The absolute and relative contribution of bacterial vitamin K to the pool used by the body is somewhat unclear [23,24].

There are several different forms of menaquinone (MK-1 to MK-14)[25], varying in the number of isoprenoid residues attached to the ring. All the vitamin K compounds possess a 2-methyl 1, 4-naphthoquinone ring. The functional group of the vitamin K is the naphthoquinone ring, so the mechanism of action is similar for all K vitamins[24].

Vitamin K is an essential cofactor for the carboxylation of glutamate to gamma-carboxyglutamic acid (Gla), which confers calcium-binding properties to vitamin K-dependent proteins. To date, two distinct groups of Gla-containing proteins have been identified: those involved in blood coagulation and those found in calcified tissue such as osteocalcin (bone Gla protein) and matrix Gla proteins. The role of Gla-containing proteins in the blood plasma is well understood; the function of Gla-containing proteins in calcified tissues is less clear.

The three currently known vitamin K-dependent bone proteins (osteocalcin, matrix Gla protein and protein S), are synthesized by osteoblasts. Osteocalcin (OC) is thought by some to be produced solely by bone tissue[24,26], but others have reported small amounts of OC in vascular smooth muscle cells[27] and in platelets[28]. OC is approximately 20% of the noncollagenous protein in bone[24,26]. Currently, the exact function of this protein is unclear. The carboxylated glutamic acid residues of OC facilitate calcium binding to hydroxyapatite[23], and are likely involved in bone mineralization[17,19]. However, some in vitro and knock-out gene studies suggest that OC may inhibit bone mineralization[29], and may act as a negative regulator for bone formation[18, 26]. In general, OC is thought to be a marker of bone formation in postmenopausal states[24], and is likely to be both vitamin D and vitamin K dependent[18,26].

Undercarboxylated vitamin K-dependent proteins are nonfunctional[17,24]and their presence in serum has been used as a marker of Vitamin K status[18,24,26] . The carboxylation state of OC is responsive to changes in vitamin K intake levels [30-32]. While prothrombin time is a good indicator of vitamin K status for blood coagulation, it is not sensitive enough to identify deficiency states that would affect bone health. Thus, undercarboxylated osteocalcin (UcOC) is the preferred biochemical index for assessing vitamin K status with respect to bone health[33,34] .

It is difficult to define “normal” or “abnormal” values for serum OC and/or UcOC, as the types of assays used in different laboratories vary[35,36]and results need to be standardized [36]. However, many studies have shown that UcOC concentrations increase with age, from about 20% of total OC in the third to fifth decade of life to 40-50% of total OC in the later years[30-33,37]. Increasing evidence seems to indicate that

the present recommended requirements for vitamin K (1µg/kg body weight/day), which are based on blood coagulation times, may not be sufficient to ensure that all vitamin K-dependent proteins are in their maximally carboxylated forms[17,23,24].

Epidemiologic Data on Vitamin K and Bone Health

Observational Studies

Recent data suggests a potential role for vitamin K in the bone health of postmenopausal women. A number of studies have also reported significantly lower circulating levels of vitamin K in fracture patients as compared to their age-matched counterparts [6-8]. Studies examining dietary intake of Vitamin K have reported an increased fracture risk among those in the lowest quintiles of dietary vitamin K1 intake as compared to those in the higher quintiles[9,10].

The Nurses' Health Study, a prospective cohort study of 72,327 women over a 10-year period, assessed the relationship between dietary vitamin K intake and fracture risk[9]. The median intake at baseline was 169 μg/day with a range of 41-604μg/day. Women in the lowest quintile (<109μg/day) experienced significantly higher fracture risk as compared to the third quintile (146-183μg/day). The difference between the remaining quintiles was not significant. The age-adjusted relative risk for hip fractures was 1.43 (95%CI: 1.08-1.89) for the lowest quintile as compared to the higher quintiles combined. Lower dietary vitamin K intakes were also associated with an increased incidence of hip fractures in a random subset of 335 men and 553 women (average age was 75 years) in the Framingham Heart Study[38]. Individuals in the highest quartile of vitamin K intake (median: 254 μg/day) had a significantly lower 7-year relative risk of hip fracture compared to those in the lowest quartile of intake (median: 56 μg/day). In addition, low dietary vitamin K intake was associated with low BMD in women, but not in men, in the Framingham Offspring Study[11].

Serum vitamin K concentrations appear to be strongly influenced by the polymorphism of Apo E, which plays a role in vitamin K transportation in blood. Individuals with the E4 allele have been shown to have the lowest phylloquinone concentrations[39,40]. Apo E4 has also been associated with fracture risk and low BMD in studies of Caucasian and Japanese postmenopausal women[41-44].

The effect of long-term administration of oral anticoagulants (vitamin K antagonists) on bone is controversial. While a number of studies report a significant reduction in BMD[45-48], others have not detected a similar relationship. In a meta-analysis of nine cross-sectional studies, long-term exposure to oral anticoagulants was related to a significant decrease in BMD at the ultradistal radius, but not at the lumbar spine, femoral neck or femoral trochanter[49]. The Study of Osteoporotic Fractures examined bone density and fracture rates in 149 warfarin users compared to 6052 nonusers and did not find a difference between the two groups[50]. These studies have methodological issues that make interpretation and generalization of the results difficult: most studies are cross-sectional in nature; and the participants on anticoagulants often have comorbid conditions and poorer health.

Interventional Studies

Most randomized placebo-controlled trials published to date have examined the relationship of vitamin K2 supplementation (45mg/day) to BMD or fracture incidence, and most were conducted in Japan[12-16] . The largest study followed a total of 205 osteoporotic women randomly assigned to receive either 45mg vitamin K2 (menatetrenone) plus calcium supplementation (150 mg elemental calcium/day) or calcium supplementation alone[12]. Participants were prohibited from taking drugs that could affect bone and

calcium metabolism. At the end of 2 years, the treated group experienced significantly less reduction in BMD (L2-L4) as compared to controls (-0.4±0.7% vs. -2.6±0.6%; p=0.019). As well, the incidence of clinical fractures was significantly lower in the treated group (13% vs. 30%; p=0.027). Serum levels of OC rose significantly in the treated group (42.4±6.9%) as compared to controls (18.2±6.1%), suggesting accelerated bone formation, while serum levels of UcOC were significantly lower in the treated group (1.6±0.1 ng/ml vs. 3.0±0.3ng/ml; p<0.0001).

Of the remaining studies, one examined the effect of vitamin K2 supplementation among 72 postmenopausal women[13], one examined the effect among 110 women receiving leuprolide therapy[15], one examined the effect on 108 stroke patients[16], and another examined the effect among 20 men and premenopausal women with chronic glomerulonephritis[14]. These studies all observed similar suppression of bone loss among participants receiving vitamin K2 supplementation, though not all reached statistical significance. Of the two that measured markers of bone formation, only one found a significant difference in the treated group[15].

With respect to interventions with vitamin K1, there have been few published studies to date[30-32,51]. The largest study, a randomized placebo-controlled trial involving 181 healthy postmenopausal women, compared vitamin K1 (1mg/d) plus minerals (calcium, magnesium, zinc) and vitamin D (8 μg/day) to two other groups: one with minerals and vitamin D only and one with placebo[51]. At the end of 3 years of treatment, the group receiving vitamin K1 had a 1.7% higher femoral neck BMD compared to the minerals and vitamin D only group and a 1.3% higher femoral neck BMD compared to the placebo group. However, there was no difference in spine BMD among the three groups.

Another randomized placebo-controlled trial involving 111 healthy women between the ages of 50 and 85 years examined urinary calcium loss in those who received vitamin K1 at 1mg/day compared to placebo for 3 months[31]. Women were classified as fast or normal losers of calcium based on calcium/creatinine ratios in fasting urine samples. After one month of vitamin K supplementation, the urinary calcium loss was significantly less among the fast losers as compared to their matched controls and remained significant after the third month (mean change -30%; p<0.005). There was no effect seen among the normal calcium losers over the treatment period. Among women receiving vitamin K1, levels of serum cOC increased significantly, as did bone-specific alkaline phosphatase. Levels of UcOC remained unchanged.

A third study involved 20 postmenopausal osteoporotic women with previous Colles fractures[32] . The study used a cross-over design, randomly assigning women to initially receive either vitamin K1 (1mg) or a combination of vitamin K1 (1mg) plus vitamin D (400IU) for two weeks. After a 3 month 'washout' period, assignments were reversed. After two weeks of supplementation, total serum OC levels increased in both the vitamin K1 and vitamin K1+D groups but only reached significance in the latter group. Degree of carboxylation increased significantly in both groups (p<0.001). Carboxylation percentages returned to that observed at baseline 10 weeks after supplementation stopped. Urinary calcium, creatinine and bone-specific alkaline phosphatase (BAP) levels did not differ significantly before and after supplementation.

1.5 How will the results of this trial be used?

If vitamin K1 supplementation is shown to significantly and safely decrease bone loss, it can be used in conjunction with calcium and vitamin D supplementation for the prevention of postmenopausal

osteoporosis. The results of this study will also inform recommendations for vitamin K1 requirements for optimal bone health, including the potential adverse effects of long-term vitamin K1 supplementation. Since osteopenia affects 45% of women age 50 and over in Canada, the results of this study can have a significant impact in the care of these women, and in the field of osteoporosis.

1.6 Describe any risks to the safety of participants involved in the trial.

This is a very safe study with minimal risks. The potential risks are:

1) Adverse effects from vitamin K supplementation –

Doses of 5 to 10mg of vitamin K1 have been administered safely as short-term treatment for bleeding disorders in North America. Over the past 15 years, Dr. Vermeer's group in the Netherlands has used oral doses of vitamin K1 ranging from 1mg to 10mg per day in studies of 3 to 12 months' duration without any observed adverse effects (C. Vermeer, personal communication). The Japanese groups have used up to 90mg per day of oral menaquinones (vitamin K2) for up to 2 years without any observed adverse effects as well (M. Shiraki, personal communication). The only side-effect associated with vitamin K1 supplementation that we were aware of when we submitted our original proposal is nausea. Currently, we have 435 participants in the study (77 of whom have completed 2 year follow-up) (Appendix 2A) and only 3 of the participants have complained of nausea.

2) Radiation from BMD tests --

The amount of radiation associated with a bone density test and an instant vertebral assessment (IVA) is small and is about the same as what one's exposure to the natural environment each day.

3) Risks from other procedures (e.g. blood draw) –

For most women, needle punctures for blood draws do not cause any serious problems. However, they may cause bleeding, bruising, discomfort, infections, and/or pain at the needle site or dizziness. Blood draws in this trial will be performed by study staff experienced with phlebotomy.

4) Risks of Declining BMD –

Women are discontinued from the study if their BMD falls below a T-score of –2.5 at 1 year at the lumbar spine, femoral neck or total hip.

For our trial extension (Appendix 3), we are excluding those whose BMD falls below T-score of –2.5 at 2 years at the lumbar spine, femoral neck or total hip. In addition, any woman whose BMD has dropped more than 10% at the lumbar spine, femoral neck or total hip from baseline to 2 years will be excluded from participation in the study extension.

5) Risks of Fractures –

Women will be excluded if they develop clinical fragility fractures, such as low-trauma fractures sustained from standing height, at any point in the study; these women will be referred for treatment. A committee involving two of the co-investigators (Drs. Hawker and Josse) will adjudicate all fractures.

2.0 THE PROPOSED TRIAL

2.1 What is the proposed trial design?

In our original application to CIHR in 2001, we proposed to carry out a single-centre (University of Toronto) double-blind, placebo-controlled randomized trial. This trial has been in progress over the past 3 years.

2.2 What are the planned trial interventions? Both experimental and control.

The treatment intervention is 5mg of vitamin K1 per day. This dosage was chosen because it has the highest potential for an observed therapeutic effect and short-term safety data were available for up to 10mg per day at the time of our application. Women assigned to the treatment group receive 5mg of vitamin K1 per day in the form of tablets and women assigned to the placebo group receive tablets that look and taste identical to those of the treatment group.

All women in the trial are assessed for their calcium and vitamin D intake at each visit and will receive calcium and vitamin D supplementation if they are not taking enough through their diet. We aim to achieve daily intakes levels of 1500mg of calcium and 800 iu of vitamin D, levels recommended for postmenopausal women with osteopenia.

A physician (either Dr. Cheung or Dr. Tile) will perform annual history and physical examinations on all study participants as part of the International Conference on Harmonization of Technical Requirements for Registration of Pharmaceuticals for Human Use (ICH) Good Clinical Practice guidelines.

Rationale for Selection of Dose of Vitamin K

Vitamin K is an essential cofactor for the carboxylation of glutamate to gamma-carboxyglutamic acid (Gla), and makes vitamin K-dependent proteins functional. Recent studies suggested roles for various vitamin K-dependent proteins at multiple stages of bone metabolism. To date, the three identified Gla-containing vitamin K-dependent proteins in bone are matrix Gla protein, protein S and osteocalcin. Osteocalcin is the most studied protein in bone, but it accounts for only a portion of the Gla-content in mature bone[52]. Previous studies found that 1mg of vitamin K1 (phylloquinone) is sufficient to fully carboxylate osteocalcin[30] which is responsible for the binding of calcium to hydroxyapatite[6,17,19]. Matrix Gla-protein appears earlier in calcification then osteocalcin and binds to both the organic and mineral components of bone[53]. Although osteopenia and fractures have been associated with deficiency in matrix Gla protein, the exact role of vitamin K in matrix Gla protein is still unclear. Since measuring matrix Gla protein in the laboratory proves difficult, no one knows the dose of vitamin K1 required to fully carboxylate this protein. Thus, the exact amount of vitamin K1 needed to optimize the carboxylation of all vitamin K-dependent bone proteins has not been established, but is at least 1mg per day.

Recent studies also showed that vitamin K exerts its effects on bone through pathways other than carboxylation. The side chain of menaquinone-4 (MK-4 or menatetrenone, one of the vitamin K2 homologues) was shown to have inhibitory effects on osteoclast formation, an effect which was independent of the carboxylation of osteocalcin[54]. MK-4 has been used in several randomized controlled clinical trials in Japan and was found to be effective in reducing postmenopausal bone loss[12]. Several research groups recently demonstrated that MK-4 could be formed in animal tissues from phylloquinone (vitamin K1)[55,56]. Therefore, the optimal dose of vitamin K1 for bone metabolism is not solely determined by its effects on carboxylation.

Doses of 5 to 10mg of vitamin K1 have been administered safely as short-term treatment for bleeding disorders in North America. Over the past 15 years, Dr. Vermeer's group in the Netherlands has used oral doses of vitamin K1 ranging from 1mg to 10mg per day in studies of 3 to 12 months' duration without any observed adverse effects (C. Vermeer, personal communication). The Japanese groups have used up to 90mg per day of oral menaquinones (vitamin K2) for up to 2 years without any observed adverse effects as well (M. Shiraki, personal communication).

To maximize the probability of finding a clinically and statistically significant effect of vitamin K on

BMD and minimize the risk of adverse effects of vitamin K, we chose an oral dose of 5mg per day. This dose represents the midpoint for doses used in previous studies and the maximum dose that can be put into a tablet form for easy administration (J. Elliot at Roche Vitamins Inc., personal communication).

2.3 What are the proposed practical arrangements for allocating participants to trial groups?

Participants are screened at baseline and assigned to treatment or placebo groups using a list of random numbers generated in blocks of 10. Three such lists were produced by Mr. Kenneth Milstead (MSc, Epidemiology), our database manager, and given to the research pharmacy at Toronto General Hospital, University Health Network. The chief research pharmacist, Mr. Ron Seto, then picked one of these lists and assigned either odd or even numbers as the treatment group. He also checked to see that the randomization by such means is balanced. He and his staff then labeled the study pills from 1 to 435 according to the treatment assignment in this list. Both the vitamin K pills and the placebo pills were sent directly to the research pharmacy for labeling. Consecutive successfully screened participants are given already labeled study pills (with number 1 going to our first study participant and number 435 going to our last participant). The three lists and the treatment assignment are not available to the investigators or study personnel.

2.4 What are the proposed methods for protecting against other sources of bias?

In order to minimize bias, the study is double-blinded so that the participants, the study personnel and the investigators are unaware of the participant assignments. The vitamin K and placebo pills look and taste the same. The pill bottles look the same, other than the study number. There are no adverse effects that would unblind the participant assignment. Both participants and study personnel are blinded to BMD results at 1 and 2 years (our primary outcome). Serum samples for vitamin K analyses and bone turnover markers are stored in a -80C freezer. These will be analyzed in batches and the corresponding results stored by Dr. Vieth and Dr. Thompson (both of whom have no contact with participants) until the end of the trial.

2.5 What are the planned inclusion/exclusion criteria?

Our study population is postmenopausal women with osteopenia, with T-scores between –1.0 and –2.0. We restrict our recruitment to women who are osteopenic rather than osteoporotic, because women with osteoporosis are at a significantly increased risk for fragility fractures and there are current established clinical guidelines for treating postmenopausal women with T-scores below –2 [3,4]

To be eligible for inclusion into the trial, women have to be:

1. Postmenopausal:
   - One year since the natural cessation of menses, or
   - Hysterectomy with either postmenopausal status confirmed by FSH lab values, or age 55 and above

AND

2. Osteopenic:
   - T-score at baseline has to be between (and including) -1.0 and -2.0 in the lumbar spine (L1-L4), total hip or femoral neck, and
   - the lowest reading of the above three measurements must be between -1.0 and -2.0

If women have ANY of the following exclusion criteria, they are not eligible for the trial. These criteria are to ensure safety of the participants and to enhance internal validity of the study:

1. Women ever having had a fragility fracture after age 40;
2. Women currently on anticoagulants, previously on anticoagulants in the past 3 months, or expected

to be on anticoagulants in the near future;

3. Women on hormone replacement therapy, raloxifene, bisphosphonates or calcitonin during the past 3 months;
4. **Women who have ever been on a bisphosphonate for more than 6 months,**
5. Women previously diagnosed with Paget's disease, hyperparathyroidism, hyperthyroidism or other metabolic bone diseases;
6. Women with decompensated diseases of the liver, kidney, pancreas, lung, or heart
7. Women with a history of active cancer in the past 5 years;
8. Women taking mega-doses of vitamin A (more than 10,000 iu per day) or E (more than 400 iu per day);
9. Women involved in other clinical trials;
10. Any women who, in the opinion of the principal investigator, is at poor medical or psychiatric risk for the study.

2.6 What is the planned duration of the treatment?

In our original application, we proposed to treat all participants for a total of two years from baseline randomization. As of August 30, 2004, 435 have been randomized and 77 have completed 24-month follow-up assessment (Appendix 2.1). Based on our current schedule, all participants will have completed 24-month follow-up by August 2006 (instead of our original estimation of February 2005).

ECKO trial extension

We will capitalize on the longer study length by incorporating a new extension phase in individuals who have completed their 24-month follow-up assessment (ECKO trial extension). This extension will provide valuable safety data on the long-term use of vitamin K supplementation in postmenopausal women with osteopenia as well as long term fracture data. Individuals who have completed their 24-month visit will be invited to continue in this ECKO trial extension for 1-2 years. They will be asked to continue to take study pills and have every 6-month follow-up, beginning at their 24-month visit and extending until the time point at which the last recruited study participant completes her 24-month visit (August 2006) (Appendix 1.2B, 3.1 and 3.2). We estimate that approximately 70 of our original participants will agree to participate in the 2-year extension (62 of 68 have agreed to participate so far, and we expect another 18 to complete 24-month follow-up by August 2004) and approximately 140 will agree to participate in the 1-year extension. In this subset of participants, we will examine whether the effect of vitamin K1 on spine and total hip BMD plateaus with time. As of August 2004, we have not had SAEs that are beyond our expectations for this population. Both our arms-length safety monitoring committee at McMaster's University and our Research Ethics Board have agreed to this extension (Appendix 1.1B). As we have to maintain staff until August 2006 to complete the original trial, minimal additional resources would be needed to follow a subgroup of our participants until that date.

2.7 What is the proposed frequency and duration of follow up?

In our original application**,** we proposed to have follow-up visits at 3, 6, 12, 18, 24 months after the baseline randomization visit. The trial is progressing well accordingly to protocol (see Appendix 2.1). The only minor exception is during the two SARS outbreaks in Toronto in March –April 2003 and in May-June 2003. During that period, every precaution was taken to safeguard the health of our participants. Participants scheduled for visits were contacted by telephone and either went though a telephone visit or rescheduled to a new date. Also, special arrangements were made to ensure an uninterrupted supply of study pills.

In this renewal application, we propose to have follow-up visits at 30, 36, 40 and 48 months for participants joining the ECKO trial extension (Appendix 3.1).

2.8 What are the proposed primary and secondary outcome measures?

In our original proposal, the primary outcome of interest is change in BMD. Secondary outcomes of interest are the adverse effects of long-term vitamin K1 supplementation, biochemical markers of bone turnover, degree of carboxylation of OC and health-related quality of life. Based on reviewers' comments, we have amended our proposal to include fragility fractures as a secondary outcome. This will include clinical fragility fractures as well as morphometric fractures.

2.9 How will the outcome measures be measured at follow up?

Primary Outcome:

BMD in the lumbar spine (L1-L4) and left total hip are measured by Dual Energy X-ray Absorptiometry (DEXA). An experienced technologist will use standardized methods to acquire and analyze scans using a Hologic QDR 4500 Delphi A scanner (Hologic Inc, Waltham, Massachusetts) at the University Health Network Osteoporosis Program Bone Density and Body Composition Laboratory. Recent precision studies were performed in this laboratory using the method described by Bonnick et al[57]. Thirty-two volunteers with T-scores ranging from –2.9 to 4.1 were scanned twice with on-and-off the scanning table and repositioning in between scans. Our results show coefficient of variations of 0.89 % for the lumbar spine (L1-L4) and 1.09% for the total hip, which are consistent with other published data.

Daily and quarterly quality control methods are performed in this laboratory. The quality control logs over the study period are reviewed as part of quality assessment of the bone density scans in the study. In addition, Dr. Lee, co-investigator and certified clinical densitometrist, is reviewing all scan quality and will make sure that the analyses fields are comparable for all scans. Dr. Lee has no contact with study participants and is blinded to treatment groups.

Secondary Outcomes:

Adverse effects: Adverse events are monitored throughout the trial period. Specifically, participants have been asked to inform us of any perceived change in well-being and if they feel develop a new condition, start a new drug, undergo any procedure or require hospitalization for any reason, without regard to causal relationship. We also enquire about these issues when they come for their follow-up visits. Serious adverse events (SAEs) are defined as death, any hospitalization, a life-threatening experience (that is, immediate risk of death), cancer, severe or permanent disability, or congenital anomaly, according to regulations set out by the Food and Drug Administration (FDA) and all records related to the event will be requested. Data will be captured on standardized reporting forms (included in the original proposal) and all events will be reported to the safety committee. Drs. Hawker and Josse, rheumatologist and endocrinologist (both blinded to treatment groups), will adjudicate all serious adverse events. SAEs are also sent to our arms-length safety committee (Drs. Adachi and Papaiannou) at McMaster University within 24 hours of our trial team being informed of them. They can unblind if they feel that the adverse event warrants unblinding. At the end of the study, we will analysis all reported conditions by treatment groups to assess for any possible relationship to vitamin K administration.

Up to August, 2004, we have not had any unexpected SAEs, in terms of frequency of conditions expected in this population. Our independent safety committee has reviewed our events and agreed to the continuation of the trial (Appendix 1.1C).

Biochemical markers: Serum OC will be measured using a radioimmunoassay kit (Incstar, Stillwater, Minnesota) as described by Knapen et al [30]. Serum UcOC, as a measure of vitamin K status, will be measured based on different affinities of cOC and UcOC for hydroxyapatite. Briefly, 300μL samples will be incubated with 30 mg hydroxyapatite (calcium phosphate tribasic type IV, Sigma Chemical Co) in Eppendorf tubes, mixed end-over-end for 1 hour at 4°C and then centrifuged. The supernatant will be decanted. The unbound OC remaining in the serum supernatant will be measured as above, with radioimmunoassay, and the amount of UcOC will be calculated by subtracting unbound OC from total OC. UcOC level will be expressed as a percentage of total OC concentration. The limit of detection for OC is 0.78 ug/L.
Serum levels of cross-linked N-telopeptides of type I collagen (NTx) will be measured using a microplate enzyme-linked immunosorbent assay (ELISA) in a competitive inhibition format as described by Scariano et al.[58]. This assay uses a specific monoclonal antibody (MAb1H11). Results will be reported as nanomoles of bone collagen equivalents (BCE)/L. The limit of detection is 1.0 nmol BCE/L.

Serum bone specific alkaline phosphatase (BAP) will be measured using a commercially available immunoassay kit in a microtiter strip format with a monoclonal anti-BAP antibody coated to the strips (Alkphase-B, Metra Biosystems, Mountain View, CA).

Health-Related Quality of life: In our original proposal, we proposed to use two instruments to assess changes in health-related quality of life at baseline and at 24 months -- the Medical Outcomes Survey 36-item short form questionnaire (SF-36)[59,60] and the Osteoporosis Quality of Life Questionnaire (OQLQ)[61]. However, prior to starting our study, we decided to use the SF-36 only due to time constraints and because our population is a healthy population and most women do not have back pain or restrictions to daily activities because of osteoporosis.

The Medical Outcomes Survey 36-item short form questionnaire (SF-36) has been tested for reliability and validity in the general population and in the postmenopausal population[59,60]. It assesses nine health domains: physical functioning, role functioning-physical, bodily pain, general health, vitality, social functioning, role functioning- emotional, mental health and reported health transition. The SF-36 is self-administered and can be completed in less than 15 minutes[60,62].

Fractures: Participants are asked to inform us if they sustain a fracture. In addition, on every follow-up visit, we inquire whether they have experienced a fracture since their last visit. Radiological reports will be obtained to confirm the diagnosis of clinical fractures. Dr.s Josse and Hawker will adjudicate these events and will decide whether they are fragility fractures or not.

In addition, we are performing instant vertebral assessments (IVA) using our Hologic 4500 Delphi A bone densitometer at baseline and 24 months. The IVA provides an economical and relatively low radiation alternative to lateral X-ray morphometric analysis of vertebral fractures. These scans have been used to evaluate morphometric fractures in other studies with reasonable reliability and validity[63-66]. Trained technologists will assess these scans for morphometric fractures using the quantitative and semi quantitative methods by Genant et al[67,68] .

We will assess both clinical fragility fractures and vertebral morphometric fractures as secondary outcomes. This analysis is exploratory in nature.

2.10 Will health service research issues be addressed?

In our original proposal, we included health-related quality of life measures as a secondary outcome (see Section 2.9). If vitamin K supplementation proves to be effective in slowing bone loss in postmenopausal women with osteopenia, we will submit a separate proposal to examine the cost-effectiveness of supplementing vitamin K in postmenopausal women.

2.11 What is the proposed sample size?

The sample size calculations in our original proposal (Appendix 4) were based on our primary outcome, which is the difference in the changes in spine (L1-L4) and total hip BMD (delta) between the vitamin K group and the placebo group at the end of 2 years, expressed as g/cm^2. Because of precision issues of BMD measurements, a change of less than 3% is usually not considered clinically significant. Thus, our sample size calculation is based on this minimally important difference in BMD. This magnitude is similar to the effect of calcium and vitamin D supplementation on BMD in postmenopausal women. When we used an assumption of 10% withdrawal rate (stopping study medication) per year and a 5% drop out rate (lost to follow-up) per year, we estimated that we need 200 patients per group (total of 400 patients) in order to observe a clinically meaningful and statistically significant difference between the two groups.

Since the original application, we have revised our sample size to 430 to account for expected SAEs or AEs in this population that will necessitate discontinuation of study pills or participation in the trial.

2.12 What is the planned recruitment rate?

Recruitment of 400 postmenopausal women was originally planned to take 1.5 years using multiple recruitment strategies including family practice and osteoporosis clinics, community outreach and media promotion. A table of our recruitment strategies and a chart showing their relative success rates are attached (Appendix 2.2 and 2.3). To date we have screened 6254, invited 856 to have a BMD test and successfully recruited 435 postmenopausal women. This ratio is similar to other postmenopausal health studies[69-71]

Recruitment actually took 3 years (we estimated 1.5 years in our original proposal) and was completed in August 2004. The principle explanation for this discrepancy is the two unexpected SARS outbreaks in Toronto hospitals in 2003. Prior to the SARS outbreaks in Toronto, we were randomizing on average 17 participants per month, which would have brought us to 400 participants randomized by December 2003. However, post-SARS recruitment dropped drastically. Hospitals were closed to non-essential staff from March 2003 to the end of April 2003 and again from May 2003 to June 2003. Even in the fall of 2003, there was ongoing public concern with regard to the infectious potential of being at a Toronto hospital and recruitment dropped to 6 participants per month. More recently in 2004, recruitment has rebounded to an average of 12 participants per month. A detailed outline of our recruitment strategies and our recruitment results is shown in Appendix 2.2 and 2.3.

2.13 Are there likely to be any problems with compliance?

At each follow-up visit, participants are asked if they are taking the study pill correctly and if they have any questions or issues. They are also encouraged to call us if they have issues or questions with regard to the study or the study pills. Participants are asked to return all study pills that they have not used at follow-up visits and these are counted and recorded by two separate individuals. Overall, our compliance so far is 94%. This is based on pill counts with noncompliance defined as taking less than 80% of total pills.

2.14 What is the likely rate of loss to follow-up?

We estimated a loss to follow-up rate of 5% per year, based on other trials that we have been conducting. Based on current trial data, our loss to follow-up rate is 6%, which is lower than our estimated rate.

2.15 How many centres will be involved? This is a single-centre study.

2.16 What is the proposed type of analysis?

For our primary analyses, we will analyze data based on the principle of intent to treat. Significance will be assessed at a level of α=0.05.

Baseline characteristics for each group will be summarized and tested for statistically significant differences using the Chi-square p-values for categorical data and 2 sample t-test for continuous variables. As well, secondary diagnoses not related to the study and medication use will be summarized for each treatment group at baseline and at 24 months.

In the case of withdrawals, we will ask that they return at the two year mark for final BMD and other measurements; or, if not possible or appropriate, we will attempt to get final BMD measurement and serum samples at the time of withdrawal. We will summarize patient characteristics and reasons for withdrawals by treatment group. We will also determine whether participants who withdraw from the study differ from those who remain in terms of factors relating to the primary outcome of interest.

Analysis of the primary outcome: The primary objective of this study is to determine whether vitamin K supplementation affects BMD. The outcome for this analysis will be the changes in spine (L1-L4) and total hip BMD measurement at baseline and at two years of follow-up in the two groups. This will be assessed for significance using a 2 sample t-test.

Analyses of secondary outcomes: Changes in bone formation markers and degree of carboxylation of osteocalcin will be analyzed comparing differences in means and standard deviations between groups, at baseline and at 24 months, and changes within groups. One-way analysis of variance (ANOVA) will be used to compare treatment groups. In addition, the differences between the two groups will be assessed by the 2-tailed Mann-Whitney U test.

Analysis of adverse events will consider both number and type of adverse events in each group, and will be performed using the Chi-square test and the Fischer Exact Test. Mean changes in quality of life measures will be analyzed for each domain separately, using two sample t-test.

We will finalize our analysis plans next spring. Uunblinded analyses will only be performed at the end of the study after data has been cleaned and locked.

2.17 What is the proposed frequency of analyses?

Descriptive analyses for the whole group will be performed for submission of abstracts to scientific meetings, but outcome analyses will only be conducted after the final patient has completed the 2-year trial and data has been cleaned and locked.

2.18 Are there any planned subgroup analyses?

We are planning to examine differences in the effect of vitamin K on bone density between various

groups: early postmenopausal women versus late postmenopausal women (as rates of bone loss vary between these groups), those with ApoE4 alleles versus all others (since there is decrease in vitamin K transport in women who are Apo E 4 carriers), past versus never users of HRT, and those below age 65 versus above age 65..

2.19 Has any pilot study been carried out using this design? No.

3. **TRIAL MANAGEMENT –** This was discussed in original proposal. See Appendix 5.

Reference List

1. Tenenhouse A, Joseph L, Kreiger N, Poliquin S, Murray TM, Blondeau L, Berger C, Hanley CA, Prior JC and the CaMOS Research Group. Estimation of the prevalence of low bone density in Canadian women and men using a population-specific DXA reference standard: the Canadian multicentre osteoporosis study (CaMOS). *Osteoporos Int 11:897-904 2000.*

2. Consensus development conference. Diagnosis, prophylaxis, and treatment of osteoporosis. *Am J Med 94:646-650 1993.*

3. Fulton JP. New guidelines for the prevention and treatment of osteoporosis. National Osteoporosis Foundation. *Medicine & Health, Rhode Island. 82(3):110-1 1999.*

4. Cheung AM, Feig DS, Kapral M, Diaz-Granados N, Dodin S, and the Canadian Task Force on Preventive Health Care. Prevention of osteoporosis and osteoporotic fractures in postmenopausal women: Recommendation statement from the Canadian Task Force on preventive health care. *CMAJ 170(11):1665-1667 2004.*

5. Brown JP, Josse RG, for the Scientific Advisory Council of the Osteoporosis Society of Canada. 2002 Clinical practice guidelines for the diagnosis and management of osteoporosis in Canada. *CMAJ 167(10 suppl): S1-S32 2002.*

6. Hodges SJ, Akesson K, Vergnaud P, Obrant K, Delmas PD. Circulating levels of vitamins K1 and K2 decreased in elderly women with hip fracture. *J Bone Mineral Res* 8:*1241-5 1993.*

7. Hodges SJ, Pilkington MJ, Stamp TC, Catterall A, Shearer MJ, Bitensky L *et al*. Depressed levels of circulating menaquinones in patients with osteoporotic fractures of the spine and femoral neck. *Bone 12:387-9 1991*.

8. Bitensky L, Hart JP, Catterall A, Hodges SJ, Pilkington MJ, Chayen J. Circulating vitamin K levels in patients with fractures. *J Bone Joint Surg*. *Br 70:663-4 1988.*

9. Feskanich D, Weber P, Willett WC, Rockett H, Booth SL, Colditz GA. Vitamin K intake and hip fractures in women: a prospective study. *Am J Clin Nutr 69:74-9 1999.*

10. Booth SL, Mayer J. Warfarin use and fracture risk. *Nutr Rev 58:20-2 2000.*

11. Booth SL, Broe KE, Gagnon DR, Tucker KL, Hannan MT, McLean RR, Dawson-Hughes B, Wilson PW, Cupples LA, Kiel DP. Vitamin K intake and bone mineral density in women and men. *Am J Clin Nutr 77(2):512-6 2003.*

12. Shiraki M, Shiraki Y, Aoki C, Miura M. Vitamin K2 (menatetrenone) effectively prevents fractures and sustains lumbar bone mineral density in osteoporosis. *J Bone Min Res 15(3):515-21 2000.*

13. Iwamoto I, Kosha S, Noguchi S, Murakami M, Fujino T, Douchi T *et al*. A longitudinal study of the effect of vitamin K2 on bone mineral density in postmenopausal women a comparative study

with vitamin D3 and estrogen-progestin therapy. *Maturitas 31:161-4 1999.*

14. Yonemura K, Kimura M, Miyaji T, Hishida A. Short-term effect of vitamin K administration on prednisolone-induced loss of bone mineral density in patients with chronic glomerulonephritis. *Calcif Tissue Int 66:123-8 2000.*

15. Somekawa Y, Chigughi M, Harada M, Ishibashi, T. Use of vitamin K_2 (Menatetrenone) and 1,25-dihydroxyvitamin D_3 in the prevention of bone loss induced by Leuprolide. *J Clin Endocrinol Metab 84:2700-2704 1999.*

16. Sato Y, Honda Y, Kuno H, Oizumi K. Menatetrenone ameliorates osteopenia in disuse affected limbs of vitamin D- and K-deficient stroke patients. *Bone 23:291-296 1998.*

17. Kohlmeier M, Salomon A, Saupe J, Shearer MJ. Transport of vitamin K to bone in humans. *J Nutr 126:1192S-6S 1996.*

18. Booth SL. Skeletal functions of vitamin K-dependent proteins: not just for clotting anymore. *Nutr Rev 55:282-284 1997.*

19. Weber P. Management of osteoporosis: is there a role for vitamin K? *Int J Vitam Nutr Res 67:350-6 1997.*

20. Weber P. Vitamin K and bone health. *Nutrition 17(10):880-7 2001.*

21. Zittermann A. Effects of vitamin K on calcium and bone metabolism. *Curr Opin Clin Nutri Metabol Care 4(6):483-7 2001.*

22. Bugel S. Vitamin K and bone health. *Proceed Nutri Soc 62(4):839-43 2003.*

23. Shearer MJ. Vitamin K. *The Lancet 345: 229-234 1995.*

24. Vermeer C, Schurgers LJ. A Comprehensive Review of Vitamin K and Vitamin K Antagonists. *Hematol/Oncol Clin North Amer 14:339-352 2000.*

25. New SA. Bone health: the role of micronutrients. *Br Med Bull 55:619-33 1999.*

26. Ferland G. The Vitamin K-dependent Proteins: An Update. *Nutr Rev 56:223-230 1998.*

27. Severson AR, Ingram RT, Fitzpatrick LA. Matrix proteins associated with bone calcification are present in human vascular smooth muscle cells grown in vitro. *In Vitro Cell Dev Biol Anim 31:853-857 1995.*

28. Thiede MA, Smock SL, Petersen DN, Grasser WA, Thompson DD, Nishimoto SK. Presence of messenger ribonucleic acid encoding osteocalcin, a marker of bone turnover, in bone marrow megakaryocytes and peripheral blood platelets. *Endocrinol 135: 929-937 1994.*

29. Price PA. Role of vitamin-K dependent proteins in bone metabolism. *Ann Rev Nutr 8:565-583 1988.*

30. Knapen MH, Hamulyak K, Vermeer C. The effect of vitamin K supplementation on circulating osteocalcin (bone Gla protein) and urinary calcium excretion. *Ann Intern Med 111:1001-5 1989.*

31. Knapen MH, Jie KS, Hamulyak K, Vermeer C. Vitamin K-induced changes in markers for osteoblast activity and urinary calcium loss. *Calcif Tissue Int 53:81-5 1993*.

32. Douglas AS, Robins SP, Hutchison JD, Porter RW, Stewart A, Reid DM. Carboxylation of osteocalcin in post-menopausal osteoporotic women following vitamin K and D supplementation. *Bone 17:15-20 1995.*

33. Sokoll LJ, Sadowski JA. Comparison of biochemical indexes for assessing vitamin K nutritional status in a healthy adult population. *Am J Clin Nutr 63:566-573 1996*.

34. Rucker R. Improved functional endpoints for use in vitamin K assessment: important implications for bone disease. *Am J Clin Nutr 65:883-884 1997*.

35. Binkley NC, Suttie JW. Vitamin K nutrition and osteoporosis. *J Nutr 125:1812-21 1995.*

36. Gundberg CM, Nieman SD, Abrams S, Rosen H. Vitamin K Status and Bone Health: An Analysis of Methods for Determination of Undercarboxylated Osteocalcin. *J Clin Endocrinol Metab 83:3258 1998.*

37. Szulc P, Chapuy MC, Meunier PJ, Delmas PD. Serum undercarboxylated osteocalcin is a marker of the risk of hip fracture in elderly women. *J Clin Invest 9:1769-1774 1993.*

38. Booth SL, Tucker KL, Chen H, Hannan MT, Gagnon DR, Cupples LA et al. Dietary vitamin K intakes are associated with hip fracture but not with bone mineral density in elderly men and women. *Am J Clin Nutri 71:1201-8 2000.*

39. Saupe J, Shearer MJ, Kohlmeier M. Phylloquinone transport and its influence on caroxyglutamate residues of osteocalcin in patients on maintenance hemodialysis. *Am J Clin Nutr 58:204-208 1993.*

40. Kohlmeier M, Saupe J, Drossel HJ, and Shearer MJ. Variation in phylloquinone (vitamin K1) concentrations in hemodialysis patients. *Thromb Haemost 74:1252-1254 1995.*

41. Salamone LM, Cauley JA, Zmuda J, Pasagian-Macaulay A, et al. Apolipoprotein E gene polymorphism and bone loss: estrogen status modifies the influence of apolipoprotein E on bone loss. *J Bone Miner Res 15:308-314 2000.*

42. Johnston JM, Cauley JA, Ganguli M. APOE 4 and hip fracture risk in a community-based study of older adults. *J Am Geriatr Soc 47:1342-5 1999.*

43. Cauley JA, Zmuda JM, Yaffe K, Kuller LH, Ferrell RE, Wisniewski SR et al. Apolipoprotein E polymorphism: A new genetic marker of hip fracture risk--The Study of Osteoporotic Fractures. *J Bone Miner Res 14:1175-81 1999.*

44. Shiraki M, Shiraki Y, Aoki C, Hosoi T, et al. Association of bone mineral density with

Apolipoprotein E phenotype. *J Bone Miner Res 12:1438-1445 1997.*

45. Philip WJ, Martin JC, Richardson JM, Reid DM, Webster J, Douglas AS. Decreased axial and peripheral bone density in patients taking long- term warfarin. *QJM 88:635-40 1995.*

46. Monreal M, Olive A, Lafoz E Del Rio L. Heparins, coumarin, and bone density. *Lancet 338:706 1991.*

47. Resch H, Pietschmann P, Krexner E, Willvonseder R. Decreased peripheral bone mineral content in patients under anticoagulant therapy with phenprocoumon. *Eur Heart J 12:439-441 1991.*

48. Fiore CE, Tamburino C, Foti R, et al. Reduced axial bone mineral content in patients taking oral anticoagulant. *South Med J 85:538-42 1990.*

49. Caraballo PJ, Heit JA, Atkinson EJ, Silverstein MD, O'Fallon WM, Castro MR *et al*. Long-term use of oral anticoagulants and the risk of fracture. *Arch Intern Med 159:1750-6 1999.*

50. Jamal SA, Browner WS, Bauer DC, Cummings SR. Warfarin use and risk for osteoporosis in elderly women. Study of Osteoporotic Fractures Research Group. *Ann Intern Med 128: 829-32 1998.*

51. Braam LA, Knapen MH, Geusens P, Brouns F, Hamulyak K, Gerichhausen MJ, Vermeer C. Vitamin K1 supplementation retards bone loss in postmenopausal women between 50 and 60 years of age. *Calc Tiss Intern 73(1):21-6 2003.*

52. Robey PG, Borkey AL. GLA-containing proteins. In: Osteoporosis (Ma4rcus R, Feldman D, and Lesley J, eds.), pp 142-146, Academic Press, New York, 1996.

53. Price PA, Williamson MK. Primary structure of bovine matrix Gla protein, a new vitamin K-dependent bone protein. *J Biol Chem 260:14971-5 1985.*

54. Hara K, Akiyama Y, Nakamura T, Murota S, Morita I. The inhibitory effect of vitamin K2 (menatetrenone) on bone resorption may be related to its side chain. *Bone 16(2):179-84 1995.*

55. Thijssen HH, Drittij-Reijnders MJ. Vitamin K distribution in rat tissues: dietary phylloquinone is a source of tissue menaquinone-4. *Brit J Nutr 72(3):415-25 1994.*

56. Davidson RT, Foley AL, Engelke JA, Suttie JW. Conversion of dietary phylloquinone to tissue menaquinone-4 in rats is not dependent on gut bacteria. *J Nutr 128(2):220-3, 1998.*

57. Bonnick SL. Bone Densitometry in Clinical Practice. *New Jersey: Humana Press Inc., 1998.*

58. Scariano JK, Glew RH, Bou-Serhall CE, Clemens JD, Garry PJ, Baumgartner RN. Serum levels of cross-linked N-telopeptides and aminoterminal propeptides of Type I collagen indicate low bone mineral density in elderly women. *J Bone Miner Res 23:471-477 1998.*

59. Ware JE, Sherbourne CD. The MOS 36-item Short-Form Health Survey (SF-36). I. Conceptual

framework and item selection. *Med Care 30:437 1992.*

60. McHorney CA, Ware JE, Raczek AE. The MOS 36-item Short-Form Health Survey (SF-36). II. Psychometric and clinical tests of validity in measuring physical and mental health constructs. *Med Care 31:247-263 1993.*

61. Osteoporosis Quality of Life Study Group. Measuring quality of life in women with osteoporosis. *Osteoporos Int 7:478-487 1997.*

62. McHorney CA, Ware JE, Rogers W et al. The validity and relative precision of MOS short- and long-form health status scales and Dartmouth COOP charts: results from the Medical Outcomes Study. *Med Care 30:MS253 1992.*

63. Black DM, Aren NK, Palermo L, Pearson J, Cummings SR, for the Study of Osteoporotic Fractures Research Group. Prevalent vertebral deformities predict hip fractures and new vertebral deformities but not wrist fractures. *J Bone Miner Res 14:821-828 1999.*

64. Wasnich RD. Vertebral Fracture Epidemiology. *Bone 18(3):179S-183S 1996.*

65. Greenspan SL, von Stetten E, Emond SK, Jones L, Parker RA. Instant vertebral assessment: a noninvasive dual X-ray absorptiometry technique to avoid misclassification and clinical mismanagement of osteoporosis. *J Clin Densitom 4(4):373-80 2001*.

66. REA JA, Li J, Blake GM, Steiger P, Genant HK, Fogelman I. Visual Assessment of vertebral deformity by x-ray absorptiometry: A highly predictive method to exclude vertebral deformity. *Osteopor Int 11:660-668 2000.*

67. Genant HK, Li J, CY Wu, Shepherd JA. Vertebral Fractures in Osteoporosis. A new method for clinical assessment. *J Clin Densitom 3(3):281-290 2000.*

68. Rea JA, Chen MB, Li J, Potts E, Fan B, Blake GM, Steiger P, Smith IG, Genant HK. Morphometric X-ray absorptiometry and Morphometric Radiography of the spine: A comparison of analysis precision in normal and osteoporotic subjects. *Osteopor Int 9:536-544 1999.*

69. Grady D, Applegate W, Bush T, Furberg C, Riggs B, Hulley SB for the HERS Research Group. Heart and estrogen/progestin replacement study (HERS): design, methods, and baseline characteristics. *Control Clin Trials 9:314-35 1998.*

70. Writing Group for the Women's Health Initiative Investigators. Risks and Benefits of Estrogen Plus Progestin in Healthy Postmenopausal Women: Principal Results From the Women's Health Initiative Randomized Controlled Trial. *JAMA 288:321-333 2002.*

71. Tworoger SS, Yasui Y, Ulrich CM, Nakamura H, LaCroix K, Johnston R, McTiernan A. Mailing strategies and recruitment into an intervention trial of the exercise effect on breast cancer biomarkers. *Cancer Epidemio Biomark Prev 11(1):73-77 2002.*
